# Legacy of peatland erosion shapes microbial communities during recovery

**DOI:** 10.1038/s43247-026-03766-8

**Published:** 2026-06-30

**Authors:** Fin Ring-Hrubesh, Mike Vreeken, Anne Eberle, Bradley Welch, Paul Sinnadurai, Penny Johnes, Robert Griffiths, Angela Gallego-Sala, Richard Pancost, Casey Bryce

**Affiliations:** 1 School of Earth Sciences, University of Bristol, Bristol, UK; 2 Cabot Institute for the Environment, Royal Fort House, University of Bristol, Bristol, UK; 3 Organic Geochemistry Unit, School of Chemistry, University of Bristol, Bristol, UK; 4 Bannau Brycheiniog National Park Authority, Brecon, Powys UK; 5 School of Geographical Sciences, University of Bristol, Bristol, UK; 6 School of Environmental and Natural Sciences, Bangor University, Bangor, Gwynedd UK; 7 Geography, College of Life and Environmental Sciences, University of Exeter, Exeter, UK; 8 Present Address: GFZ Helmholtz Centre for Geosciences, Potsdam, Germany

**Keywords:** Biogeochemistry, Microbial ecology, Soil microbiology

## Abstract

Human degradation has caused many peatlands worldwide to shift from long-term carbon sinks to net sources. In upland blanket peatlands, erosion disrupts plant-derived carbon input and exposes deep peat, accelerating oxidation of old carbon. The efficacy of restoration in preventing carbon loss and recovering ecosystem function depends on microbial responses to both water table manipulation and renewed litter input. Yet it is unclear how these factors alter the microbial communities that ultimately control carbon storage and emissions. Here we show that microbial community composition in the eroded Waun Fignen Felen peatland, South Wales, was governed primarily by organic matter bioavailability rather than water-table position. Long-term erosion leaves a legacy of highly decomposed organic matter, unaltered by re-wetting. Where plant litter accumulation is renewed on formerly eroded peat surfaces, the influx of bioavailable organic input supports a distinct prokaryote community with a greater bacterial population size, and evidence of elevated respiration.

## Abstract (web summary)

Microbial community composition is primarily governed by the renewal of plant litter at the peat surface and utilization of recent bioavailable organic matter in the recovering layer, rather than water-table position, based on field study at Waun Fignen Felen peatland, South Wales, UK

## Introduction

Northern peatlands operate as a large terrestrial carbon store (400–600 GtC; refs. ^1–3^). |In the UK, peatlands cover 8–12% of the terrestrial surface with ombrotrophic upland bog being the dominant peatland type (65% of peatland area)^4^. Peatland surface erosion and vegetation loss are common facets of upland blanket bog degradation^5^. Vegetation loss can be driven by several factors, including excessive grazing pressure^6^, die-off due to intolerance of atmospheric pollutant and nutrient deposition^7,8^, and fire^9,10^. As vegetation acts to stabilise the peatland surface, its disappearance can lead to enhanced erosion by surface water flow, wind action and frost-heave^11–13^. Vegetation loss can contribute to peatland surface drying, particularly through reduced cover of *Sphagnum* mosses, the principal vegetation in undisturbed northern ombrotrophic bogs, which play a key role in retaining surface water^14^. A positive feedback may also develop whereby enhanced rates of erosion, and the hostile microclimate, and desiccation of bare peat surfaces hinder the re-colonisation of vegetation^6,15^, such that bare peat surfaces persist along with associated carbon losses.

Peatland carbon can be lost as both a direct and indirect result of erosional processes. Organic matter is washed out from the peat profile directly, entering watercourses as particulate organic matter with detrimental consequences for water quality^16^, and further decomposing in streams to dissolved organic carbon (DOC) and CO_2_^17^. In severely eroded UK blanket peatlands, Pawson et al.^18^ observed that carbon losses through fluvial export alone total 29–106 MgC km^−2^ yr^−1^. Stored carbon is also destabilised as an indirect consequence of erosion, where gully formation drives water table drawdown and enhanced mineralisation in the deepened oxic layer^19^. Peatland drying leads to increased CO_2_ emissions and a suppression of CH_4_ emissions with a net warming effect overall^20,21^. Although impacts of drying on DOC production and export are reported, there is greater uncertainty over the direction of the response^22–24^, with DOC representing a heterogeneous pool of microbial and plant metabolites governed by a wide range of biotic and abiotic processes. To address both direct and indirect carbon loss pathways, restoration of eroded peatlands will typically aim to stabilise peat surfaces and re-establish vegetation, while also implementing re-wetting measures.

Typically, the composition of peatland organic matter follows a depth gradient, with deep peat dominated by less reactive organic compounds, such as lignin and polyphenols, which have persisted over time^25^. While the anaerobic decomposition of these molecular classes does occur, it proceeds slowly, leading to their relative persistence^26^. Conversely, recently deposited near-surface peat will contain more “labile” organics such as polysaccharides, which are typically more microbially available^27^. In a system where surface peat has been lost, the contrast in the composition of shallow and deep organic matter could be lessened or even absent^28^.

The impact of altering the physico-chemical environment through concurrent re-wetting and vegetation re-establishment on peatland microbial communities remains uncertain. Understanding the microbiome response is of major relevance given its central role in the peatland carbon cycle and ecosystem function^29,30^. The primary aim of this work is to determine the relative influence of substrate supply, bulk peat organic matter composition and water-table position on microbial community composition during peatland restoration. Where restoration occurs, this will typically proceed from a degraded baseline, and reduced microbial activity has been reported in more mineralised peats in several studies^28,31,32^. Other studies have found organic matter quality to be strongly associated with microbial community shifts^33,34^, though these investigations did not make comparisons with the relative effect of water table. Given the widely-reported controlling effect of water table position^29,35^, we hypothesised that this would be a stronger predictor of microbial community composition during peatland recovery.

We examine microbial communities at the upland ombrotrophic bog of Waun Fignen Felen in South Wales. The site serves as a case study due to the co-location of areas representing long- and short-term regeneration atop erosional surfaces, alongside ongoing surface erosion. We show that differences in bulk peat organic matter composition are strongest across depth profiles where peat is actively accumulating. Where long-term recovery has occurred, this surface layer supports a microbial community distinct from both the underlying deep peat microbiome and that in degraded surface peats exposed by erosion. We find that during recovery from erosion, the switch to supply of recent substrate input from vegetation, superimposed onto otherwise low-bioavailability bulk peat can exert an even stronger influence on microbial community composition than differences in water table position, contrary to our initial hypothesis.

## Results and discussion

### Water table regimes align with regeneration status

**Fig. 1:**
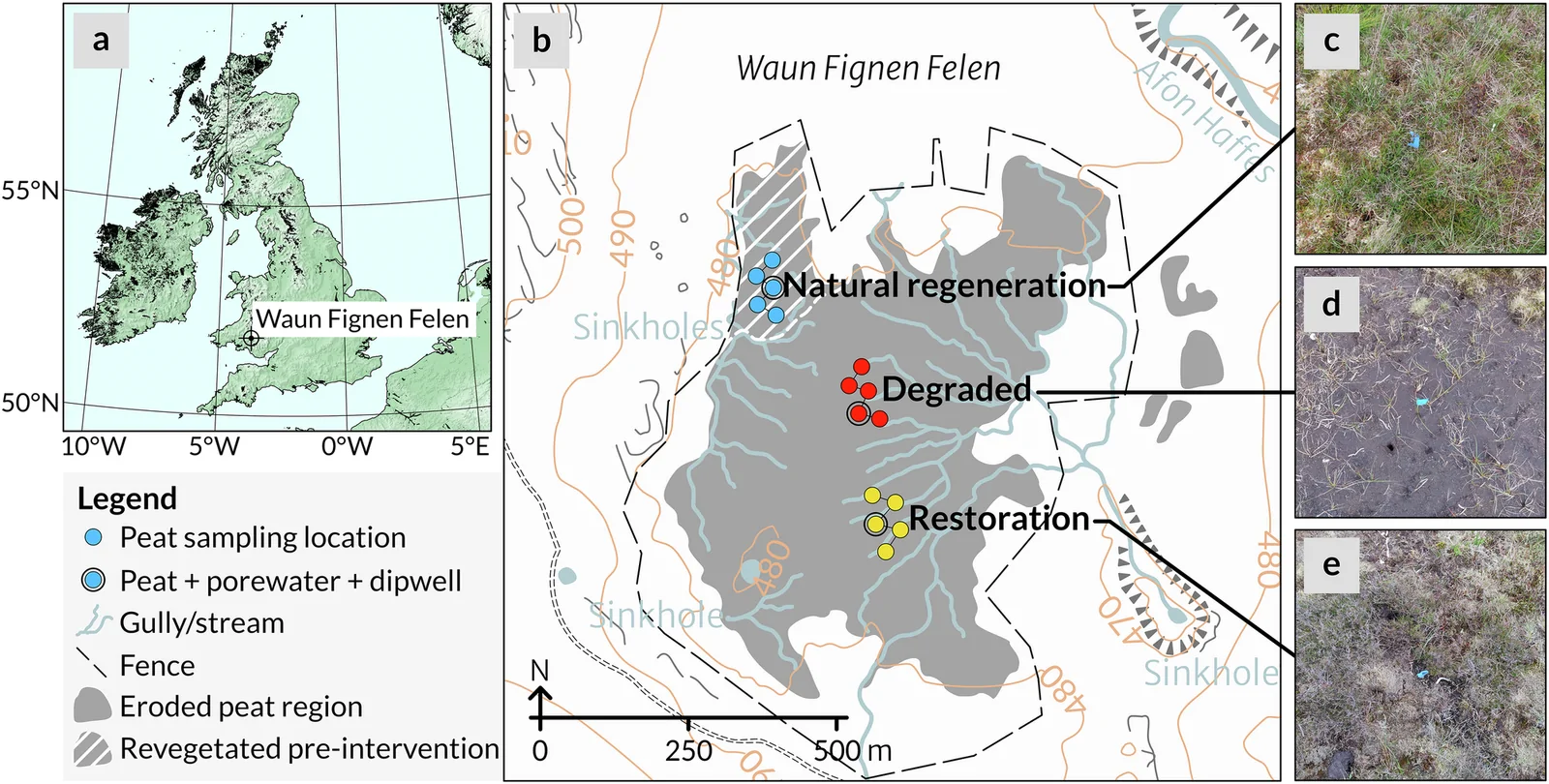
Site overview and sampling scheme. **a** Location of Waun Fignen Felen, estimated peatland regions are indicated by black shading, these are derived from the PEATMAP project^92^. **b** Locations of peat coring (filled points) and of dipwells and porewater sampling (points with additional circle) within the fenced study region at Waun Fignen Felen (see legend for symbology). Red points represent degraded areas, yellow represent restoration and blue represent natural regeneration. **c–e** Photographs of the peat surface at the sampling locations; each image covers approximately 1 × 1 m of the ground surface.

This study examined three hydrologically independent areas within the region of severe historic erosion at Waun Fignen Felen which each represent contrasting states of recovery (Fig. 1). These were (1) a “degraded” area which was actively eroding with a predominantly bare surface; (2) an area of “managed restoration” which has become re-vegetated following intervention to block adjacent gullies in 2009; and (3) an area of “natural regeneration” in which vegetation has naturally recolonised a former erosional surface, with aerial photographs confirming recolonisation by 1990 at the latest (detailed site information can be found in the “Methods” section).

**Fig. 2:**
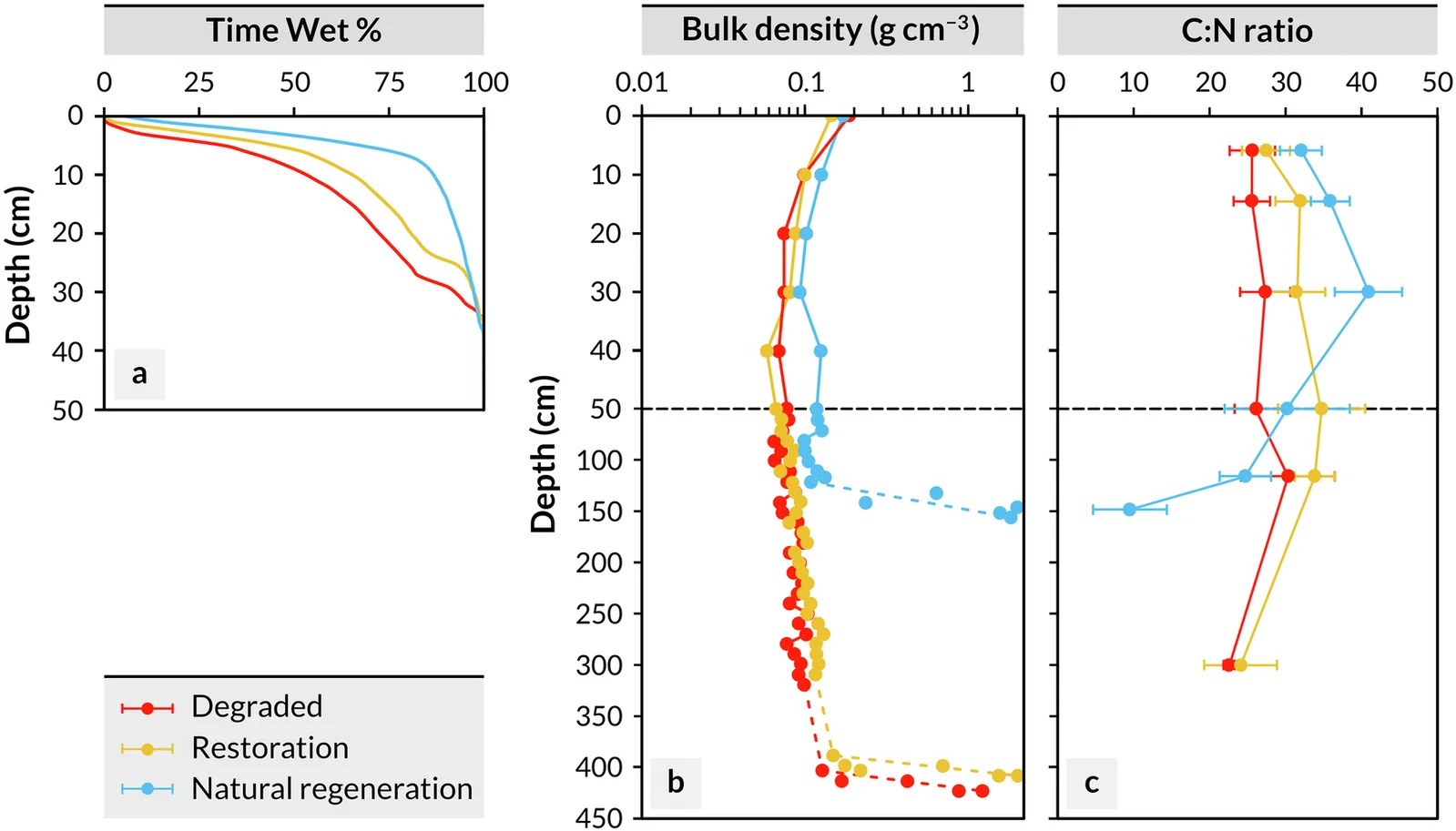
Summary of peat properties across the three conditions. **a** The proportion of time for which peat depths were below the water table during the two week period prior to core sampling, below 50 cm (*y*-axis limit) peat was permanently inundated during the entire monitoring period. **b** The peat bulk density (dry mass/in situ volume) at 10 cm intervals, presented along a log-scale due to steep changes at the peat base. Values were derived from the core taken at the porewater sampling location. A solid line is used to connect datapoints; near the peat base a dashed line is used to approximate the trend. **c** The carbon-to-nitrogen (C:N) ratio of bulk peat across all cores, with error bars representing the standard deviation. For depths 6, 14, and 50 cm *n* = 15 (3 replicate cores at 5 locations); for depths 30, 105 and 147/300 cm *n* = 3 (3 locations). Note change in y-axis scale at 50 cm. In all three panels, red is used for the degraded peat area, yellow is used for the restoration and blue is used to represent the natural regeneration.

Over the two-year monitoring period, water tables were consistently highest in the natural regeneration site, intermediate in the managed restoration site and lowest in the degraded site (Fig. 2). This trend, aligning with the degradation status, was magnified by more rapid drops in water table in the degraded and restoration areas during periods of low precipitation (Supplementary Fig. B). None of the site water tables were extremely deep, with mean water table in the degraded peat at 12.9 ± 10.1 cm below the surface, compared to 9.7 ± 8.9 cm in the restoration profile, and 5.5 ± 7.2 cm in the naturally regenerating area ( ± standard deviations). Across the monitoring period Waun Fignen Felen received >2400 mm of annual rainfall. This is considerably above the minimum typically associated with upland blanket peatland (1600 mm^36^). The high water tables across the whole site are likely supported by this high annual precipitation as well as the relatively flat topography of the eroded site, conferring lower rates of runoff.

### Extent of recovery evident in geochemical profiles

There was evidence for substantial historic surface peat loss at all three study areas within the peatland, with the degraded profile continuing to undergo active erosion. By contrast, recent accumulation was visibly apparent in the top 3–5 cm of the restoration profiles and the upper 5–15 cm in the natural regeneration area. Despite this, the bulk density of surface peat was comparable across the three conditions. The shallower peat base in the natural regeneration site was evident in the bulk density profiles (rapid increase associated with the peat base; Fig. 2), and elevated bulk density values were seen in this profile between 40 and 110 cm relative to the degraded and restoration areas.

Natural regeneration was associated with a surface layer that had the highest C:N ratio. In the degraded and restoration areas, lower C:N ratios at 6, 14, and 30 cm corresponded with the absent or thin accumulating layers in these profiles, suggesting that shallow peat in these areas is more decomposed than in the natural regeneration region, as differential loss of C during decomposition has been reported widely^37,38^. Beneath the natural regeneration surface layer, there was a transition to amorphous peat, which was reflected in the steep decline in C:N ratios in this profile compared to consistent values downcore in the other areas (Fig. 2). Correspondingly, linear mixed models indicated that degradation status (*F*_2,160_ = 33.37), depth (*F*_2,160_ = 22.33) and their interaction term (*F*_2,160_ = 8.67), all had a significant effect on the C:N ratio (*p* < 0.001).

While the peat profile in the naturally regenerating area may have been somewhat shallower due to its location further from the centre of the raised peat dome, the differences in peat physical properties also indicate that prior erosion may have been more advanced in this area, having exposed older deep peat before the onset of natural recovery. Regeneration atop erosional horizons, has been described in other UK peatlands^39,40^, with Milner et al.^40^ setting out the case whereby exposure of an erosion-resistant layer allows the system to transition to a new stable state in which renewed peat accumulation can proceed. At Waun Fignen Felen, exposure of peat layer with a higher bulk density and lower hydraulic conductivity (Supplementary Fig. C) may have facilitated a comparable transition in the region of natural regeneration.

**Fig. 3:**
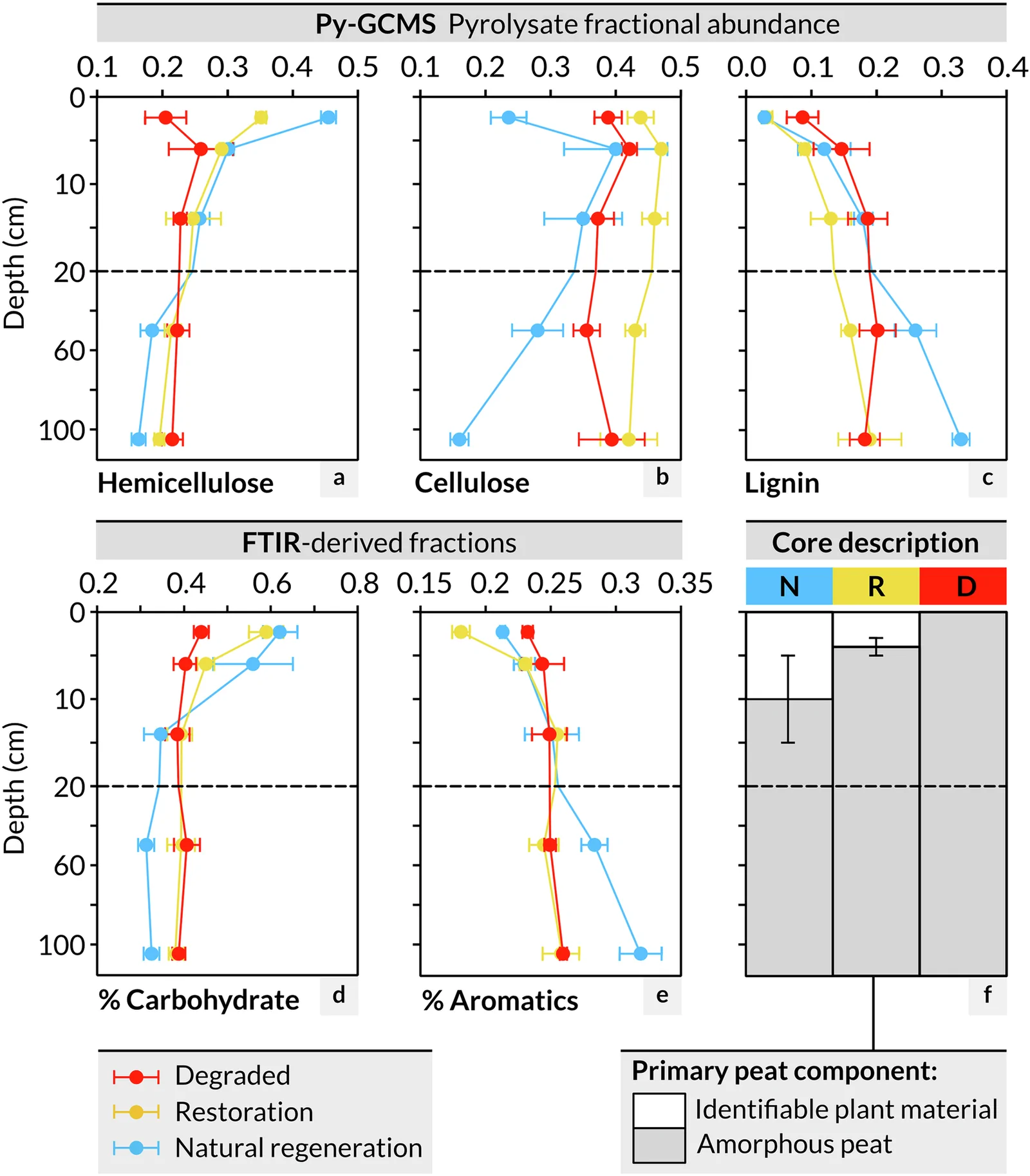
Peat organic matter composition. **a**–**c** A comparison of relative abundances of organic macromolecular groups in bulk peat as derived by Py-GC-MS (determined from integrated peak areas of relevant pyrolysis product major ions as in Schellekens et al.^78^; values given as a proportion of all identified peaks for qualitative assessment). **d**, **e** FTIR-derived organic matter fractions (relevant bands processed using the calibrated method set out by Hodgkins et al.^83^ to provide semi-quantitative values) from triplicate samples collected in summer 2022. Note change in *y*-axis scale at 20 cm. Error bars for Py-GC-MS and FTIR-derived fractions represent the standard deviation between samples. Red is used to represent the degraded area, yellow the restoration area, and blue the natural regeneration area. **f** An overview of the position of the erosional horizon within profiles, with error bars indicating the observed range in position.

Peat macromolecular composition, as assessed using Py-GC-MS and FTIR, also reflected the recovery status, driven by a combination of historical factors, including burial depth and erosion (Fig. 3). In shallow samples the central role of fresh plant-derived input was apparent, with a greater proportion of labile, polysaccharide-derived compounds present in the vegetated areas. In addition, the polysaccharide pool within shallow samples from vegetated profiles contained a greater relative contribution from hemicellulose-derived pyrolysis products than cellulose (Fig. 3), indicating fresh accumulation of plant material. Linear mixed modelling supports the interpretation of a degradation status effect on the hemicellulose: cellulose ratio (*F*_2,37_ = 6.91, *p* = 0.003). A depth (or status × depth) effect on this ratio was not present across the entire depth profile used in the linear mixed model (Supplementary Table 1), with this fresh material signature limited to only the nearest surface samples. By contrast, markers of lignin and other phenolics (and aromatic signals in FTIR, representing approximately the same pool) were depleted in the shallow peat, especially at the two revegetated sites. All these observations are consistent with the contemporary regrowth of fresh vegetation (primarily mosses and graminoids) at the natural regeneration site.

With increased depth, the proportion of pyrolysates associated with lignin, as well as the aromatic FTIR-derived signal, increased (Fig. 3). Most polysaccharides decompose preferentially to lignin, such that the lignin: polysaccharide ratio was strongly affected by depth (linear mixed modelling; *F*_1,35_ = 177.26, *p* < 0.001) with downcore relative lignin-enrichment. This is expected with downcore degradation of peat organic matter, but we suggest that this trend primarily reflects the switch from fresh vegetation to more strongly decomposed peat below the erosion horizon. This would explain the weaker downcore change at the degraded bare surface sites where fresh vegetation is absent (Fig. 3). The increase in lignin/aromatic components with depth at the natural regeneration sites was particularly steep, so much so that their proportions were greater than at the degraded and restored sites in the deepest peat (Fig. 3), reflecting the decline in C:N. Given that all three sampling locations were sited in the same basin, with comparable dates of peat initiation across this area set out by Smith and Cloutman^41^; the shallower peat depth now present in the natural regeneration area; and greater inferred erosional loss; we suggest that the high aromatic content reflects older peat exposed in that area, potentially with different, more lignin-rich vegetation inputs (greater contribution from vascular plants with depth; for example in Biester et al.^42^).

**Fig. 4:**
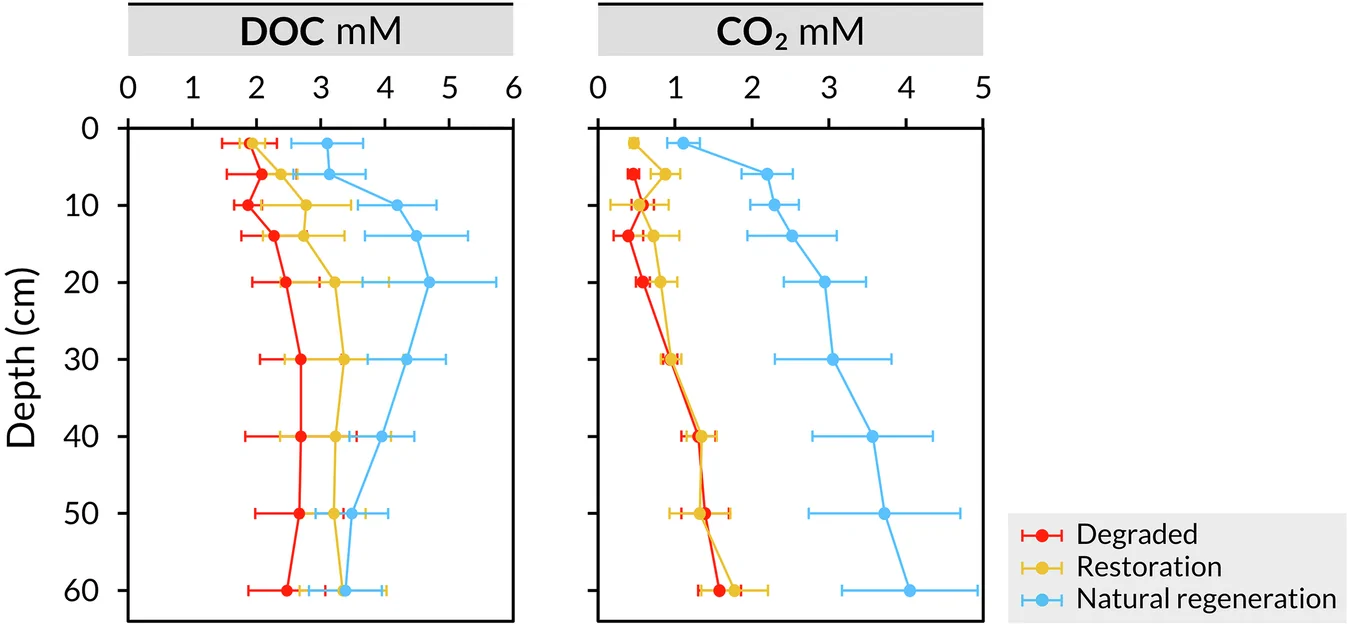
Depth profiles of dissolved organic carbon and carbon dioxide in porewaters at Waun Fignen Felen. Dissolved organic carbon was sampled on 12 occasions between August 2021–October 2022, CO_2_ samples were collected using the headspace equilibration method on 4 occasions between May–October 2022. Points represent mean values with standard deviation shown by bars. Red is used to represent the degraded area, yellow the restoration area, and blue the natural regeneration area.

Degradation status had a strong, significant effect on DOC concentration (LMM: *F*_2,237_ = 40.58, *p* < 0.001), with porewater DOC notably lower in the degraded area than either of the re-vegetated areas (Fig. 4). Studies on DOC release after peatland restoration report highly contrasting outcomes, and when reviewing available data Evans et al.^22^ found only limited support for any decrease associated with restoration, and ref. ^43^ show no consistent effect. In cases such as ours where apparent DOC production is instead lower in degraded sites than under recovery, several explanations have been suggested: Comparing eroded and intact peatland sites in the Pennines, Evans et al.^44^ attributed lower DOC export from eroded sites to either lower “lability” or minimal interaction between water and the peat matrix. Elsewhere a recovery of plant productivity has been reported to cause increased porewater DOC^45^, while Pinsonneault et al.^46^ demonstrate higher water extraction of DOC from fresh *Sphagnum* and vascular plant material than from litter or peat material. Bernard-Jannin et al.^24^ report that DOC export did not decrease under restoration, instead being sustained by a supply of more recent organic matter at the peat surface. We found that where highly aromatic macropolymers comprised a greater proportion of the organic substrate, this was associated with lower accumulation of dissolved organic compounds in porewater. In the naturally regenerating area, where porewater DOC was highest, there was a temporally consistent peak at a depth of 14–20 cm (Fig. 4). The peak in DOC concentrations in the near-surface, and association between higher concentrations and areas with vegetated peat surfaces, suggest that it originates from modern plant matter through litter or root exudates. This would represent a more accessible pool of potential substrates for microbial metabolism than DOC derived from the bulk peat organic matter^46^.

Correspondingly, dissolved CO₂ was elevated in the region of natural regeneration throughout the downcore profile, despite the higher water table in this profile, whereas CO₂ concentrations were low under enhanced degradation, pointing to the lower availability of the remaining organic matter. This effect of degradation status was supported by the linear mixed model (*F*_2,71_ = 80.35, *p* < 0.001). Our interpretation that elevated CO_2_ under the re-vegetated scenario predominantly results from the cycling of modern plant-derived carbon, would imply that this is not detrimental to the long-term carbon store. Yet this would need to be confirmed by further investigation of the peatland carbon balance, particularly as priming of microbial communities by labile carbon input has been proposed to enhance peat decomposition^47–49^. Our combined data suggest that re-vegetation may act as an overriding control on CO_2_ production, with restoration adding labile inputs that are superimposed onto low bioavailability bulk peat in formerly-eroded peatlands. No similar effect was observed with regard to methane concentrations, which were similar between the conditions (Supplementary Fig. D).

### Microbial community composition is vertically stratified

**Fig. 5:**
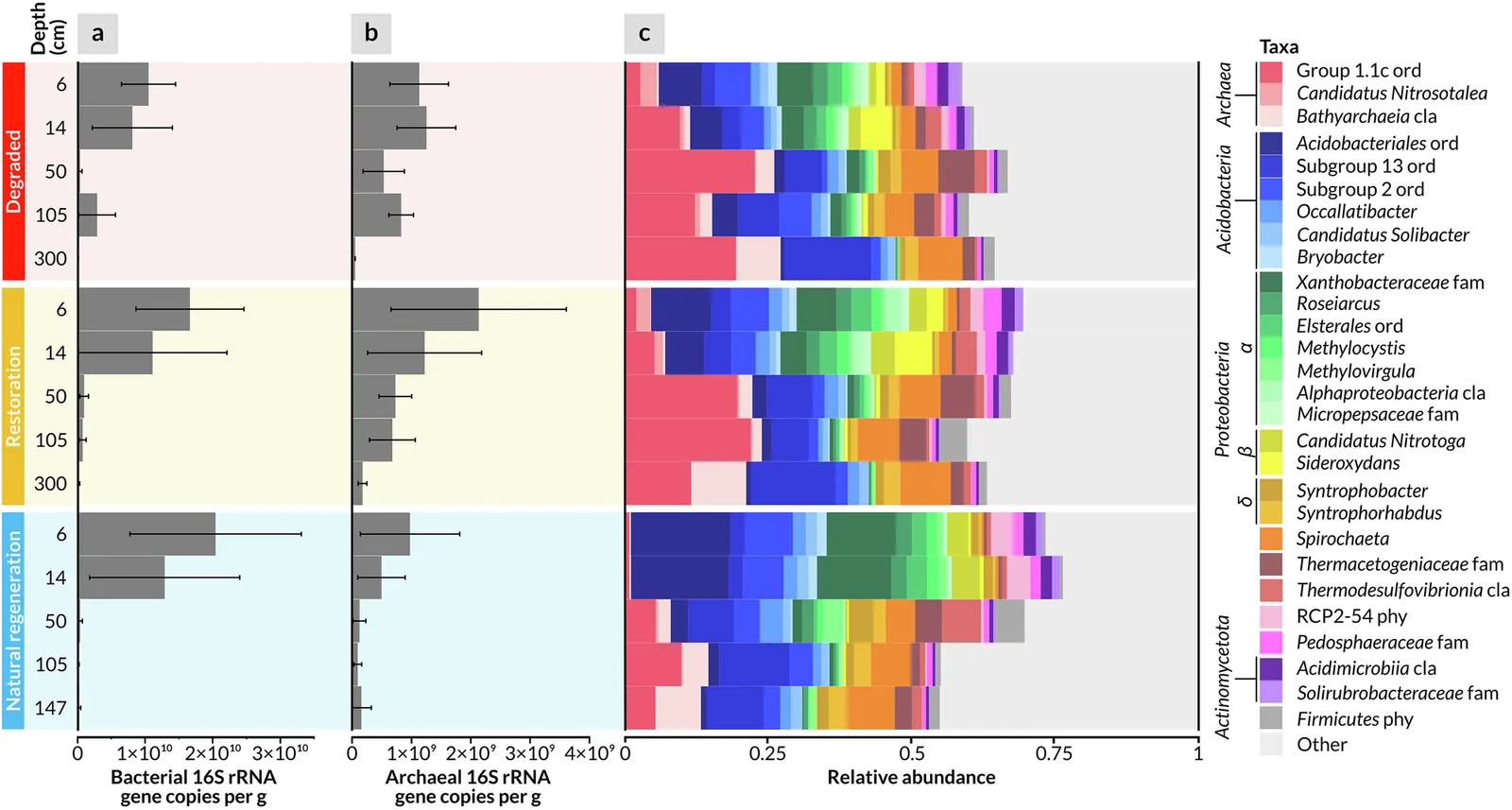
Prokaryote communities at Waun Fignen Felen. **a, b** Bacterial and archaeal abundance derived by qPCR across the depths sampled. Population sizes are approximated by quantification of 16S rRNA gene copies, with values shown as gene copies per gram of dry peat, error bars represent the standard deviation between samples. **c** 16S-derived community composition. Mean relative abundance of the most abundant 28 taxa across all samples with taxa ordered by phylum (colours in legend). Where identification at the genus-level was not possible the taxonomic rank presented is indicated by a three letter abbreviation in the legend. For each condition, *n* = 15 at depths of 6, 14 and 50 cm, and *n* = 3 at 105 cm and basal (147/300 cm) depths.

In the upper part of the profiles (6 and 14 cm), the natural regeneration area hosted a greater bacterial abundance, as assessed by qPCR, than either the bare or restoration peat profiles (Fig. 5a). Total archaeal abundance was lower throughout the natural regeneration depth profiles compared to the degraded and restoration areas (Fig. 5b). In shallow peat, the microbial community composition in the natural regeneration locations was distinct from that in the degraded and restored areas (Fig. 5c), with a markedly greater abundance of members of the order *Acidobacteriales* and family *Xanthobacteraceae*, and lower abundances of Group 1.1c *Thaumarchaeota* as well as *Sideroxydans* and *Spirochaeta*.

Acidobacterial sequences are frequently among the most abundant in peatlands, with *Acidobacteriales* one of two orders which are dominant in acidic bogs globally (alongside *Bryobacterales*, which were less abundant here)^30,50,51^. A dominance of *Acidobacteriales* in the surface litter layer and subsequent decline in deeper peat has also been reported by Golovchenko et al.^52^ in pine swamp peatland. The order includes both aerobic and anaerobic acidophilic chemoorganoheterotrophs, known to degrade a wide range of mono-, di-, and polysaccharides (Dedysh and Oren, 2020). The other taxon which was more abundant in the natural regeneration area, was the *Xanthobacteraceae*, elsewhere found to be common among the acrotelm microbiome^52–54^. These are chemoorganoheterotrophs with some strains capable of sulfur oxidation and others able to fix nitrogen^55^. By contrast, the near-surface of the managed-restoration and degraded areas was enriched in the genera *Sideroxydans* and *Candidatus Nitrosotalea* (Fig. 5c), both of which are known chemolithotrophs. These differences in the community composition, as well as abundance, correspond with the finding of greater resource-availability in the naturally regenerating area due to a greater abundance of labile organics relative to the peat everywhere else at the site.

**Fig. 6:**
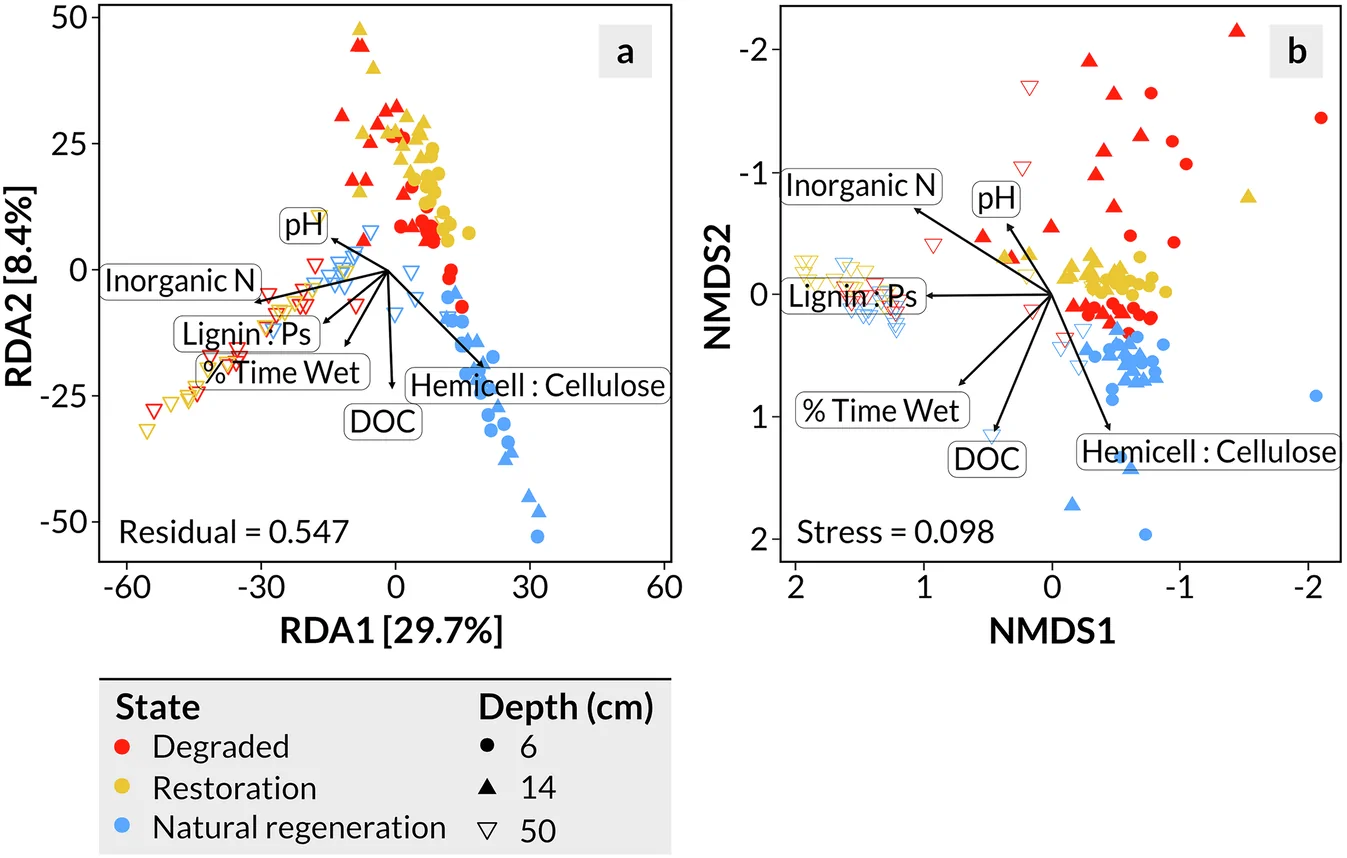
Geochemical drivers of the microbial community. **a** Redundancy analysis (RDA) with geochemical parameters that contributed significantly to the explained variation in the relative abundance of amplicon sequence variants (ASVs) between samples, as derived by 16S gene metabarcoding. Axis labels show the proportion of variation explained by each canonical axis. **b** Non-metric multidimensional scaling (NMDS) based on distance matrices computed from ASV abundances using the Bray–Curtis method. The same environmental variables selected for the RDA are fitted to the unconstrained ordination using the envfit function in the vegan R package. In both panels, red points are used to represent the degraded area, yellow the restoration area, and blue the natural regeneration area. For each degradation status and depth there is a sample size of *n* = 15.

The composition of the microbial community began to converge in samples retrieved from below 50 cm and clustered closely in deeper samples, where peat composition and conditions were similar across the three conditions (Fig. 6). This was accompanied by a decline in bacterial and archaeal 16S gene copies in all the sites, relative to the upper profile, with low total counts in all three locations below 50 cm (Fig. 5a, b). This is likely to reflect reduced prokaryote abundance in these deep peats. The deep peat community was enriched in members of the genus *Spirochaeta*, which contains obligate and facultative anaerobes involved in saccharolytic fermentation^56^. Also more abundant were the *Thaumarchaeota*, of which members described to date have been chemolithoautotrophic with some additionally capable of utilising simple organics heterotrophically^57^. In the deepest samples, proximal to the peat base, an increase in relative abundance of *Bathyarchaeota* was observed. Archaea from this phylum could be key degraders of organic matter in peatlands particularly at depth, given their similar dominance in deep marine cores^58^ and the diverse strategies for carbon assimilation that have been attributed to them, ranging from lignin metabolism to methanogenic CO_2_ uptake^59,60^. These findings support the inference that the lower rates of heterotrophic microbial activity observed in deep and highly decomposed peats^32,61,62^, reflect the lower bioavailability of remaining substrate.

Many of the taxa associated with deep peat also dominated in the shallow degraded and restoration sites, and as a result the vertical change in community abundance was weaker in those profiles (Fig. 5c). At these depths, peat was permanently inundated at all locations, although the organic matter composition diverged among them (Fig. 3), primarily due to the relatively elevated content of aromatic polymeric material at depth in the natural regeneration profile.

### Organic matter quality shapes microbial community structure

To assess the relevance of the geochemical parameters measured in this study on the observed community composition, redundancy analysis (RDA) was employed after selection of the strongest explanatory variables as detailed previously. To assess the role of water table position, the proportion of time for which each sampling depth was below the measured water level (“time wet”) was calculated over the preceding 2 weeks. Water level position was not parameterised directly as this is both highly intercorrelated with other depth-related parameters (including lignin proportion, dissolved ammonium and pH), and is less closely related to oxygen availability, the mechanism by which wetting is primarily expected to impact microbes. There was greater difference for time wet among the three sites than was derived from the dip to water level.

The RDA (Fig. 6a) reflected the convergence of the community at depth, with the greatest spread in relation to factor 1. The contrasting communities in the shallow peat were also represented in the RDA with minimal overlap, primarily explained by factor 2. This clustering was the same as that present in non-constrained ordination using non-metric multidimensional scaling (NMDS; Fig. 6b), indicating that the environmental factors included could account for much of this difference. After sequential removal of explanatory variables, the 6 primary environmental factors included in the RDA were the hemicellulose: cellulose ratio, dissolved inorganic *N* concentration, lignin: polysaccharide ratio, DOC concentration, pH and proportion of time below the water table (Fig. 6a). While all other parameters have previously been discussed in detail; the pH differed according to degradation status (LMM; *F*_2,154_ = 12.09, *p* < 0.001), being marginally elevated in the degraded profile, but did not show a depth relationship; whereas inorganic *N* content showed a depth effect (LMM; *F*_1,57_ = 64.08, *p* < 0.001) with higher concentrations in deeper peat principally due to elevated ammonium (Supplementary Fig. E).

The proportion of time below the water table, accounted for less variance than either of the variables relating to peat organic geochemistry. The shallow community under natural regeneration clustered separately along the RDA2 axis, associated most strongly with differences in the hemicellulose: cellulose ratio and DOC concentration. At 50 cm the communities of the three states clustered more closely and were differentiated from the shallow peat community by a spread along the RDA1 axis. This depth-related spread was strongly associated with dissolved inorganic *N* and the ratio of lignin: polysaccharides. Depth trends in dissolved inorganic *N* across the site were predominantly derived from elevated ammonium and not oxidised *N* species (nitrate and nitrite; Supplementary Fig. E) at depth. As a result of the strong depth trend, dissolved inorganic *N* was the strongest predictor of variance identified (Table 1), but was of limited relevance to the treatment (between-state) effect observed in shallow peat. In unconstrained ordination (NMDS), clustering according to degradation history was also observed, and where the same environmental factors are fitted, a comparable relationship with community composition can be seen (Fig. 6b).

**Table 1:** Results of ANOVA from redundancy analysis (RDA)

Parameter	Variance (%)	*F*-statistic	*p*-value
Dissolved inorganic *N*	12.6	23.7	0.001
Hemicellulose: Cellulose	8.1	15.2	0.001
Lignin: Polysaccharide	4.1	7.6	0.002
DOC	3.8	7.2	0.001
pH	1.8	3.3	0.021
Time wet %	1.7	3.2	0.014

Assessment of variables which significantly influenced community composition. Percentage variance explained by each variable was calculated relative to the total inertia of the RDA model. Significance was assessed using permutation ANOVA, with pseudo-F statistics reported, all parameters are statistically significant at the *p* < 0.05 threshold.

The time that peat layers were below the water table did not emerge as a strong driver (Table 1), although this might have been expected given the dominant role of water table in regulating oxygen availability and thus microbial processes^21,63,64^. Here, the depth trends in the composition of the microbial community were largely decoupled from the water table differences. If water table was to act as the primary control, the more frequent and deeper drying of the upper profiles in the degraded and restoration areas would be expected to support a stronger vertical gradient in prokaryote community composition. Instead, the natural-regeneration profiles displayed a more marked contrast between the communities of shallow and deep peat despite more stable inundation throughout the profile, with the shallow to deep contrast instead corresponding to the boundary of accumulating and old peat.

RDA indicated that in shallow peat, differences between the community composition (spread along RDA2; Fig. 6a), were most strongly explained by the hemicellulose: cellulose ratio, an indicator of a higher supply of relatively fresh plant substrate. Elsewhere the enzyme activity of peatland decomposer communities has been shown to be more sensitive to the quality of surface litter than to water table drawdown^65^. Here we found that community composition followed a similar trend, though in our case, the specific contrast was between an absence of litter in the degraded area, a thin layer of vegetation under recent restoration (3–5 cm) and a thicker layer (5–15 cm) in the area of natural regeneration. Other studies have similarly found bare peat to host a distinct community relative to long-term vegetated comparisons, and to support lower levels of microbial biomass and activity^66,67^. We suggest that the higher lability of surface material may sustain the higher prokaryote abundance observed in the upper profile under natural regeneration, while CO_2_ concentrations of more than double those observed in the degraded and restoration areas were another indication of greater microbial respiration. Again, microbial growth in the degraded peat is apparently restricted by factors other than water table position, differences in which were small at the site.

Past mineralisation likely caused the enhanced relative stability of the deep and erosionally exposed peat, with the residual organic matter being less reactive due to the greater proportion of aromatic compounds. Lower respiration and decomposition rates^28,32,61^, enzyme activity^62^, reduced microbial biomass^31,68^ have been attributed to poor substrate quality in more highly decomposed peats, due to a higher aromatic content. Here we find the tight clustering of the deep peat microbiome (Fig. 6) to be strongly aligned with geochemical parameters (dissolved inorganic *N* content, and lignin: polysaccharide ratio), rather than the water table. Furthermore, in those profiles without or with only a thin regenerated layer, dissolved CO_2_ was lower than observed under natural regeneration, which may reflect a lower basal rate of respiration where the peat substrate is of limited availability. Although we examined dissolved CO_2_ concentration and not emission, such findings have consequences for the stability and continued release of peat carbon after restoration, and therefore our interpretation of restoration success. They suggest that in systems where past carbon loss was extensive while water tables remain high, the rate of microbial carbon utilisation would be limited as a consequence of altered organic matter quality. It should be noted that this is not evidence of a barrier to future degradation, or a resilience of peatlands to complete remineralisation, as proposed priming effects^47–49^, or more extreme drainage than in the peatland studied here could still have the potential to lift the constraints on degradation of otherwise “recalcitrant” compounds^69^.

## Conclusions

This study investigated differential recovery on former erosional horizons, a widespread feature of UK upland peatlands, by examining the site at Waun Fignen Felen, South Wales. Differences in water table position were consistent with degradation history, yet played only a limited role in shaping the microbiome. Instead, the renewal of plant litter input at the peat surface was the predominant control on microbial community composition, with communities in the recovering layer expected to utilise this more bioavailable recent organic matter. These findings highlight the importance of vegetation re-establishment for microbiome recovery and demonstrate that restoration does not necessarily involve a simple upward migration of microbes tracking water-table position. Rather, deep peat communities remained distinct, reflecting the highly decomposed nature of this peat material. The vertical stratification of microbial communities also suggests that microbiome recovery on formerly eroded surfaces may require longer-term accumulation of peat material (over decadal timescales in this study), or else the active transplant of plant communities. Re-vegetation of bare peat, and the associated carbon inputs, are required to tip the peatland carbon balance towards accumulation, although the potential for enhanced microbial processing of the newly available substrates highlights the dynamic nature of the response.

## Methods

### Site overview

The study site, Waun Fignen Felen, is an ombrotrophic bog situated on a limestone escarpment south of Fan Hir, South Wales (475 m AOD; 51.8465, −3.7103). The peat stratigraphy in the region of the peatland under investigation was characterised by Smith and Cloutman^41^, who describe *Sphagnum* spp. peat forming a raised mound overlying *Phragmites* spp. peat deposited during earlier reed-swamp conditions atop late Devensian lake sediments. The entire study region is differentiated from the blanket peat in the surrounding landscape by more severe erosion, greater peat depth and by radial drainage outwards from its centre. In their study, Smith and Cloutman^41^ reported conventional radiocarbon dates for five near-surface (< 10 cm) samples from across this eroded region, of between 4850 ± 70 and 3800 ± 70 BP (uncalibrated^41,70^), indicating that erosion had exposed ancient peat. The eroded region was bare by the 1940s (earliest record is from aerial reconnaissance flights^71^), and though the exact cause of erosional onset is not known, a technical report on the condition of the site concludes that the most likely factors for the onset of erosion were grazing pressure and the deposition of atmospheric pollutants^72^.

The presence of ongoing erosion alongside areas representing short- and long-term regeneration within the same peatland complex was a key factor in site selection. As such, we can ascribe the primary differences in geochemistry to the degree of recovery from severe erosion. All three areas of the peatland examined in this study were situated within the eroded region identified by Smith and Cloutman^41^. In the degraded area, smooth and planar surfaces of bare peat, comprised of amorphous material which was re-suspended during inundation, indicated that the surface material was actively being eroded and re-deposited. In the area of managed restoration, the dominant plants were *Deschampsia flexuosa*, *Hypnum jutlandicum* and *Campylopus introflexus*. This assemblage is unlikely to reflect that which was present during previous phases peat accumulation, with *Sphagnum* mosses absent despite their macrofossil presence within the peat profile. Management intervention in this area consisted of blocking erosional gullies with heather bales and spreading heather brash across the adjacent bare peat surfaces. As of 2022, the bales had themselves become vegetated and trapped peat sediment, and would impede surface water flow such that pools form upslope after rainfall events. In the naturally regenerating area, *Eriophorum angustifolium*, *Sphagnum* species (*capillifolium, fimbriatum, palustre*) and occasional stands of *Molinia cerulea* were the primary vegetation cover (Supplementary Fig. A). Although the palaeorecord would indicate greater *Sphagnum* spp. dominance, the assemblage observed reflects the dominant genera described at other sites in the region by Chambers et al.^73^, who ascribe increased cover by Eriophorum and Molinia to landscape-scale human disturbance. In these naturally recolonised profiles, an abrupt transition between amorphous, dark peat and an upper layer predominantly composed of identifiable plant material marked the inferred former erosional horizon. Measured peat depths in the degraded and restoration areas were 3.8–4.3 m, compared to 1.2–2.2 m in the area of natural-regeneration. The shallower peat in the latter area resulting from its position away from the centre of the peatland (basal dates from Smith and Cloutman^41^ did not indicate a younger age) and may have been further diminished by a greater extent of historic peat loss in this area which was examined further in this study.

### Water table monitoring and porewater sampling

In each study area water table position was monitored in a centrally located dip-well. Water table data was collected using pressure transducers calibrated against ambient barometric pressure at the site (HOBO U20L-01). The dip-wells were not anchored to underlying geology, and any vertical shift was recorded and manually re-set on a 6-weekly basis, with a linear correction applied to the intervening data when shifts were observed. As such, water level depths are reported relative to the peat surface. Summary rainfall data at the site (daily totals) were recorded using a “tipping-bucket”-type gauge (Ecowitt WH5360).

Porewater was sampled from three porewater membrane-exchange equilibrator (PWE) samplers each installed 2 m apart (such that *n* = 3 for each depth and condition). PWE samplers for the collection of filtered porewater were built according to the specification of Bowes et al.^74^, itself modelled on a sampler design proposed by Hesslein et al.^75^. These samplers consisted of a PVC housing that seals a series of horizontal cells behind a polyether sulfone (PES) membrane (0.2 μm pore size, Pall Life Sciences HT-200). The cells were then connected on either side to tubing, which was routed to the surface while the samplers were installed within the peat. Tubing for water sample collection was connected to the cell base, while the top of each cell was connected to N_2_-filled syringes during sampling to maintain an anoxic headspace. After sampling, the wells were refilled with N_2_-sparged de-ionised water. Samples were collected from depths of 6, 14, 30, and 50 cm at 6-week intervals between August 2021–October 2022, representing repeated measurements from the same sampling locations across time. This interval allowed sufficient time for equilibration between porewater and PWE wells between each sampling event. The samples collected were filtered with 0.2 μm PES syringe filters into N_2_-flushed vials and transferred to cold storage at 5 °C within 12 h prior to analysis.

### Porewater geochemical analyses

Porewater pH was recorded within 12 h using a calibrated probe (Thermo Scientific Orion ROSS Ultra). DOC concentrations were determined within 72 h using the non-purgeable organic carbon method (Shimadzu TOC-L). Select dissolved inorganic ions were determined by spectrophotometric methods (NH_4_^+^-N, total NO_3_^–^-N + NO_2_^–^-N, PO_4_^3^^–^-P, SO_4_^2^^−^-S using Thermo Scientific Discrete Industrial Analyzer System Reagents for Water/Environmental Samples; total dissolved Fe by the ferrozine method), with only those selected for inclusion in the RDA model presented below. Dissolved CO_2_ and CH_4_ were determined during 4 of the sampling events (2022: 19th May, 6th July, 26th August, 11th October) using the widely applied headspace-equilibration method^76^. Briefly, immediately after the anoxic sampling of porewater from the equilibrator samplers into a syringe, an equal volume of N_2_ gas was drawn from a gas-tight bag before the sample was shaken vigorously with the headspace to equilibrate for 60 s. Headspace was then transferred into a vacuum vial (Exetainer) and stored at room temperature until analysis. Concentrations in the headspace samples were determined using gas chromatography (Agilent 7890A), equipped with a flame ionisation detector (for CH_4_, and CO_2_ after methanisation to CH_4_). Dissolved concentrations in the original porewater were determined by application of Henry’s Law using water temperatures recorded by the dipwell loggers, with Henry’s Law constants and temperature dependence coefficients sourced from the NIST database^77^.

### Peat sampling

Peat cores were independent samples, collected in summer 2022 (27th June–1st July), with cores taken using a D-section (Russian type) corer. Within each of the three study areas, a nested sampling strategy was employed with 5 peat sampling locations spaced 20 m apart along a “W”-shape transect, the central position of which was co-located with the dipwell and porewater sampling (Fig. 1). In the restoration area, each sampling location was sited 2 m away from a blocked gully. Sampling of the upper 50 cm at each position was conducted in triplicate at all 5 transect positions, while cores of the full peat profile (to base) were taken only at the central 3 transect positions. All peat was transferred onto dry ice in the field prior to freezer storage at −20 °C within 12 h. Additionally, cores were subsampled in the field using sterile tools to retrieve 4 cm long subsamples, at depths centred on 6, 14, 50, 105 cm, and the peat base, which were transferred into centrifuge tubes for DNA analysis and stored at −80 °C on return to the laboratory.

### Peat geochemical analyses

Peat geochemical datasets were derived from cores from the central 3 transect positions in each area (*n* = 3 for each depth and condition; Fig. 1). The composition of organic macromolecules in the bulk peat was investigated using pyrolysis-gas chromatography-mass spectrometry (Py-GC-MS), at the same depths at which DNA sequencing was carried out. For this analysis, 0.5 ± 0.1 mg of peat was taken from the freeze-dried, and homogenised subsamples. Peat in furnaced quartz tubes was subjected to an initial thermal desorption step at 300 °C for 2 min, to remove free lipids that may obscure the lignocellulosic signal. The desorbed peat was subsequently pyrolysed at 610 °C for 20 s using a Chemical Data System 6200 series pyroprobe with the trap at 50 °C. The pyrolysates were released post-pyrolysis by heating to 300 °C for 4 min, and were transferred at 310 °C to a gas chromatograph (GC; Thermo Scientific Trace 1310) fitted with an Rtx-1 column (dimethyl polysiloxane, non-polar, 60 m × 0.32 mm ID, film 0.25 µm). The GC oven was set to heat from 40 °C (held initially for 4 min) to 300 °C at 40 °C min^−1^, with helium used as the carrier gas. The GC was coupled with a single quadrupole mass spectrometer (Thermo Scientific ISQ7000), with the detector scanning from *m/z* 50–650. Peak identification was carried out in XCalibur (Thermo Scientific) using inhouse libraries and literature. The relative abundances of components were determined from the peak areas from integrated extracted ion chromatograms of pyrolysis product major ions as in Schellekens et al.^78^, and are reported as a proportion of the sum of all those identified. The compound classes of Schellekens et al.^78^ were also used to group products, with additional components classed according to Chen et al.^79^ (2,5-Dimethylfuran) and Nowakowski et al.^80^ (Desaspidiniol). These fractional abundances enable comparison between the relative abundances of compound classes, which is inherently qualitative^81^. Within the polysaccharides, levoglucosan was used to represent the relative abundance of cellulose as it is the primary pyrolysis product^82^; all other polysaccharide pyrolysis products were grouped to represent the hemicellulose fraction. Lignin was represented functionally through phenol, guaiacol and syringol pyrolysates, while other compound classes identified were the aliphatics (alkane/alkene doublets), N-containing compounds, and aromatics. These additional compound classes affect the relative abundances of the discussed classes, but not the ratios between them.

Fourier-transform infrared spectrometry (FTIR) was conducted complementarily on the same samples to analyse the quality of the peat organic matter. FTIR spectra were obtained by averaging 16 scans of the 4000 to 600 cm^−1^ region (4 cm^−1^ resolution, 1 cm^−1^ data interval; PerkinElmer Spectrum Two). Freeze-dried and homogenized samples were placed on an ATR crystal with pressure applied to attain even coverage and contact. An automatic background correction was applied to all samples using the instrument software. Relevant bands were subsequently identified and processed using the method set out by Hodgkins et al.^83^ to provide semi-quantitative %-aromatic and %-carbohydrate values. In that study, the aromatic fraction was calibrated against Klason lignin (acid-insoluble material minus ash), and the carbohydrate fraction against total cellulose + hemicellulose (determined by acid hydrolysis and HPLC following toluene and ethanol rinses). Additionally, the total carbon and nitrogen content of the pyrolysis/FTIR subsamples was determined using an elemental analyser calibrated with reference standard NC Soil 341506 (EA; Elementar Vario MACRO Cube), with C:N ratios computed using these mass fractions. Cores taken at the porewater sampling locations were subsampled at 10 cm intervals for determination of peat bulk density profiles by drying a known volume of sample at 105 °C.

### DNA extraction, qPCR and 16S amplicon sequencing

DNA extraction was carried out from subsamples on all cores collected (such that for each depth and condition; *n* = 15 at 6, 14 and 50 cm and; *n* = 3 at 105 and basal depths). Subsamples were stored at −70 °C and were handled in a laminar flow hood, with a homogenised sample extracted across the whole 4 cm depth interval by scouring a groove along the frozen sample with a fine spatula. This scoured peat material (*ca*. 500 mg) was processed using DNeasy PowerSoil Pro extractions kits (QIAGEN).

To determine the total bacterial and archaeal abundance in peat samples, DNA extracts were used as the template for quantitative PCR (qPCR) of 16S rRNA genes. Bacterial 16S was targeted using the primers Eub338 (5′-ACTCCTACGGGAGGCAGCAG-3′) and Eub518 (5′-ATTACCGCGGCTGCTGG-3′)^84^, while archaeal 16S was targeted with the primers 915F (5′- AGGAATTGGCGGGGGAGCAC-3′) and 1059R (5′-GCCATGCACCWCCTCT-3′)^85^. Reactions were carried out on an AriaMx realtime PCR system (Agilent Technologies), with mixtures containing forward and reverse primers at 0.5 μM, iTAq Universal SYBR Green Supermix (Bio-Rad) at 1× concentration, and template DNA in a working range of 1–5 ng μL^–1^. PCR conditions for bacterial enumeration were an activation step of 2 min at 50 °C, followed by 2 min at 95 °C, then 45 3-step cycles of denaturing at 95 °C for 15 s, annealing at 55 °C for 15 s and elongation at 72 °C for 60 s. For archaeal enumeration, conditions were a 5 min initiation step at 94 °C, followed by 45 2-step cycles of denaturing at 94 °C for 10 s and 60 °C for 30 s. Melt curve analysis confirmed the fluorescence signal originated from specific PCR products. Quantification was performed using a standard curve derived from custom gBlock standards (Integrated DNA Technology) prepared with 16S rRNA genes from *Acidobacterium capsulatum* (accession no. D26171.1) and *Methanosarcina mazei* (AB065296.1) for bacterial and archaeal enumeration, respectively. qPCR efficiency was between 91.8 and 107.5% (*R*² > 0.99), abundance is reported as copies of target gene per gram of dry peat.

For 16S amplicon sequencing to describe the microbial community, PCR amplification, library preparation and sequencing were carried out commercially by Novogene (Cambridge, UK). The V4 region of the 16S rRNA gene was targeted using the Earth Microbiome Project^86^ primers 515F (5′-GTGCCAGCMGCCGCGGTAA-3′) and 806R (5′-GGACTACHVGGGTWTCTAAT-3′), before PCR products of the correct size were selected by gel electrophoresis. These primers were selected as they were designed as universal primers to target both bacteria and archaea and have been widely applied^87^. An equal amount of PCR products was pooled from each sample and end-repaired, A-tailed and further ligated with Illumina adapters. Libraries were then sequenced on an Illumina MiSeq platform to generate 250 bp paired-end raw reads.

After sequencing, the DADA2 algorithm^88^ was used to distinguish amplicon sequence variants (ASVs) and their relative abundance within each sample, using standard settings for trimming and quality filtering. Assignment of taxonomy was carried out using the SILVA database (v.138.1)^89^. ASVs which were assigned to chloroplasts and mitochondria were removed, and the sequence tables were randomly rarefied to an even size using the phyloseq *R* package^90^.

### Statistical analysis

Linear mixed models (LMMs) were used to test the response of the principal geochemical parameters to the two fixed factors of degradation status and depth, and their interaction. Replicate ID was included as a random effect to account for replicated sampling design, and sampling date was included as an additional random effect for porewater variables measured across multiple campaigns. LMMs were fitted in *R* using the package lme4. Model residuals were assessed for normality and homoscedasticity, and no transformations were applied to improve fit. Significance of fixed effects was assessed using type III analysis of variance (*F*-tests) with Satterthwaite approximations for degrees of freedom implemented with lmerTest. For LMMs, all hypothesis tests were two-sided. Marginal and conditional *R*² values were calculated using the package performance. Variables selected for the RDA (see below) or otherwise presented in figures were included in LMMs. In the text, *F*-statistics are reported along with the number of degrees of freedom and denominator degrees of freedom, and the associated *p*-value. No formal correction for multiple comparisons was applied, as analyses were hypothesis-driven and models were specified a priori. Those LMM results that are not set out in the text can be found in Supplementary Table A, with corresponding effect sizes (*β* coefficients and 95% confidence intervals) of fixed effects in Supplementary Table B.

To determine the key environmental controls on the microbial community, RDA was applied across the depths for which highly replicated sequencing data, as well as porewater samples, were retrieved (6, 14, 50 cm). Porewater (annual mean) and peat geochemical parameters were used as explanatory variables, while greenhouse gases were excluded as they were considered equally likely to be response variables. To avoid overrepresentation of multivariate geochemistry datasets, dimensionality reduction was carried out whereby the summary ratios of polysaccharides: lignin and hemicellulose: cellulose were used to represent the bulk organic geochemistry (as determined by Py-GC-MS), rather than each compound or compound class; while ammonium and oxides of nitrogen were grouped as “dissolved inorganic N”. To identify a parsimonious set of environmental variables associated with variation in microbial community composition (ASV counts), explanatory variables were first screened for multicollinearity and subsequently refined through stepwise selection. Multicollinearity was assessed using variance inflation factors (VIFs) of each variable when used as RDA constraints, and redundant variables were removed until those remaining had low VIF scores (VIF < 5)^91^. Subsequently, stepwise selection of variables was performed using the ordistep function in the vegan *R* package applying both forward and backward selection to allow iterative re-evaluation of variable inclusion. The RDA was implemented using the microViz package and is plotted with type 2 scaling to visualise the effects of explanatory variables.

The significance of variables associated with community composition (those included in the RDA) was evaluated using analysis of variance (ANOVA), with parameters considered statistically significant at the *p* < 0.05 threshold. To assess whether groupings identified through constrained ordination by RDA were universally supported, beta-diversity was also assessed by NMDS based on a Bray–Curtis dissimilarity matrix. Both the ANOVA and NMDS were implemented using the vegan *R* package.

## Supplementary information

Transparent Peer Review file

Supplementary information

## Data Availability

All data displayed in Figs. 2–5b, and raw geochemical datasets are available at Zenodo (https://doi.org/10.5281/zenodo.17368979). Raw sequencing data have been deposited at NCBI in the Sequence Read Archive (SRA) under BioProject accession number PRJNA1346434.
