# Supplementary material for: Legacy of peatland erosion shapes microbial communities during recovery
Source: Commun Earth Environ. 2026 Jun 30;7(1):838. doi: 10.1038/s43247-026-03766-8 (PMC13683034; doi:10.1038/s43247-026-03766-8)
Supplement: Supplementary file 1 [file 43247_2026_3766_MOESM1_ESM.pdf]

nature portfolio

Peer Review File

Legacy of peatland erosion shapes microbial communities during recovery

Corresponding Author: Dr Fin Ring-Hrubesh

This file contains all editorial decision letters in order by version, followed by all author rebuttals in order by version.

Attachments originally included by the reviewers as part of their assessment can be found at the end of this file.

Version 0:

Decision Letter:

** Please ensure you delete the link to your author home page in this e-mail if you wish to forward it to your coauthors **

Dear Dr Ring-Hrubesh,

Your manuscript titled "Legacy of peatland erosion shapes microbial communities during recovery" has now been seen by 3 reviewers, and we include their comments at the end of this message. They find your work of interest, but some important points are raised. We are interested in the possibility of publishing your study in Communications Earth & Environment, but would like to consider your responses to these concerns and assess a revised manuscript before we make a final decision on publication.

We therefore invite you to revise and resubmit your manuscript, along with a point-by-point response that takes into account the points raised. Please highlight all changes in the manuscript text file.

Please submit your point-by-point responses as a separate file, distinct from your cover letter where you can add responses to the Editors' comments that you do not want to be made available to the reviewers. Word files are preferred. We recommend that any figures, tables or graphs that are included in the response to reviewers are also included in the main article or Supplementary Information.

We are committed to providing a fair and constructive peer-review process. Please don't hesitate to contact us if you wish to discuss the revision in more detail.

Please use the following link to submit your revised manuscript, point-by-point response to the referees' comments (which should be in a separate document to any cover letter), a tracked-changes version of the manuscript (as a PDF file) and the completed checklist:
Link Redacted
** This url links to your confidential home page and associated information about manuscripts you may have submitted or be reviewing for us. If you wish to forward this email to co-authors, please delete the link to your homepage first **

We hope to receive your revised paper within six weeks; please let us know if you aren't able to submit it within this time so that we can discuss how best to proceed. If we don't hear from you, and the revision process takes significantly longer, we may close your file. In this event, we will still be happy to reconsider your paper at a later date, as long as nothing similar has been accepted for publication at Communications Earth & Environment or published elsewhere in the meantime.

Please do not hesitate to contact us if you have any questions or would like to discuss these revisions further. We look forward to seeing the revised manuscript and thank you for the opportunity to review your work.

Best regards,

Somaparna Ghosh, PhD
Associate Editor,
Communications Earth & Environment

Consulting Editor,
Communications Sustainability

EDITORIAL POLICIES AND FORMATTING

We ask that you ensure your manuscript complies with our editorial policies. Please ensure that the following formatting requirements are met, and any checklist relevant to your research is completed and uploaded as a Related Manuscript file type with the revised article.

For Manuscripts that fall into the following fields:
• Behavioural and social science
• Ecological, evolutionary & environmental sciences
• Life sciences
An updated and completed version of our Reporting Summary must be uploaded with the revised manuscript
You can download the form here:
https://www.nature.com/documents/nr-reporting-summary.pdf

Furthermore, please align your manuscript with our format requirements, which are summarized on the following checklist:
Communications Earth & Environment formatting checklist

and also in our style and formatting guide Communications Earth & Environment formatting guide .

*** DATA: Communications Earth & Environment endorses the principles of the Enabling FAIR data project (http://www.copdess.org/enabling-fair-data-project/). We ask authors to make the data that support their conclusions available in permanent, publically accessible data repositories. (Please contact the editor if you are unable to make your data available).

All Communications Earth & Environment manuscripts must include a section titled "Data Availability" at the end of the Methods section or main text (if no Methods). More information on this policy, is available at http://www.nature.com/authors/policies/data/data-availability-statements-data-citations.pdf.

In particular, the Data availability statement should include:
- Unique identifiers (such as DOIs and hyperlinks for datasets in public repositories)
- Accession codes where appropriate
- If applicable, a statement regarding data available with restrictions
- If a dataset has a Digital Object Identifier (DOI) as its unique identifier, we strongly encourage including this in the Reference list and citing the dataset in the Data Availability Statement.

DATA SOURCES: All new data associated with the paper should be placed in a persistent repository where they can be freely and enduringly accessed. We recommend submitting the data to discipline-specific, community-recognized repositories, where possible and a list of recommended repositories is provided at http://www.nature.com/sdata/policies/repositories.

If a community resource is unavailable, data can be submitted to generalist repositories such as figshare or Dryad Digital Repository. Please provide a unique identifier for the data (for example a DOI or a permanent URL) in the data availability statement, if possible. If the repository does not provide identifiers, we encourage authors to supply the search terms that will return the data. For data that have been obtained from publically available sources, please provide a URL and the specific data product name in the data availability statement. Data with a DOI should be further cited in the methods reference section.

Please refer to our data policies at http://www.nature.com/authors/policies/availability.html.

REVIEWER COMMENTS:

Reviewer #1 (Remarks to the Author):

Review for Communications Earth & Environment Journal
The considered manuscript entitled “Legacy of peatland erosion shapes microbial communities during recovery” presents unique findings that expands our knowledge on how microorganisms adapt to differing ecological circumstances in a damaged peatland that is undergoing partial recovery. However, while I believe these results merits publication, some minor revisions should be made. I suggest minor revisions as my comments are editorial and do not require further experimentation or data generation.
Here are my line-by-line comments:

Line 20 - I recommend rewording this first sentence. It somewhat reads that “all peatlands are now C sources.”
Line 33 – In your first couple of paragraphs, I feel it is important to discuss vegetation types/cover. Are these eroded sites once Sphagnum dominated? What are the consequences of Sphagnum loss vs vascular plant loss (as well as loss of both)? Does vascular plant or Sphagnum moss contribute more to erosion and vis versa. I feel this is important to discuss as you mention many of these sites are ombrotrophic bogs.
Line 41 – can establish
Line 42 – would “re-colonization” be more appropriate?
Line 43 – provide an appropriate citation
Line 77 – It would be good to describe what lead to the erosion scenarios in this peatland. You discussed the many ways this can occur in the previous paragraphs, but this specific circumstance should be made clear here. If it is not currently known, then that should be stated.
Line 77 & 114 – In reference to the area of managed restoration, this restoration practice should be briefly described, even while referencing another paper. This could be explained in a bit more detail in the methods as well.
Line 91 – I feel this methods section could use more detail. You have a dynamic system you’re investigating, so providing the reader with as much information as possible is important here. What are the detailed differences between your three areas of the peatland complex?
Line 114 – Do you have an idea as to why there is an area where natural restoration is occurring and not at the actively eroding area?
Line 90 (methods) – Is there any data on hand, or recent studies that have surveyed any nearby peatlands that have not been affected or eroded? While I believe the study is good enough to stand on with the site selected, it would be very beneficial to have a relatively untouched reference peatland especially to compare vegetation communities.
Line 150-154 – looks like a different font or font size.
Line 218 – Can “wet chemistry methods” be explained in more detail?
Line 232 – I would be very cautious about this interpretation. I do not agree that this can be used as a proxy for biomass. There is too much variability when extracting DNA from environmental samples, particularly peat (humic acids etc.). It would be safer to suggest that this is the amount of estimated/potentially extractable DNA from each sample or something of that nature. If you can find other studies that have used this as a proxy, that would help your argument but even then, I would be highly cautious with this.
Line 248 – Be sure that every statistical application used in this study is described here. For example, you employ NMDS in your results but do not mention it here.
Line 322 – What kind of vegetation regrowth primarily? Sphagnum and/or vascular plants?
Line 336 (Fig. 3 caption) – I would briefly explain how you calculated your fractional abundance.
Line 374 – please see previous note on this interpretation of biomass.
Line 398 – please see previous note on this interpretation of biomass.
Line 417 (Fig. 5) – mentioning or showing where the water-table is situated (average or at time of sample collection) would be useful here. A personal opinion. Please provide n=?.
Line 500 – I feel your conclusion could be stronger. You already have a long Results and Discussion section explaining your data and your conclusion could bring forth the ‘bigger picture’ of your study. What does your data suggest about the ecological functioning of these sites, now and in the future? How are you linking microbial community changes and ‘recovery?’

Reviewer #2 (Remarks to the Author):

This is a fine case study of a peatland with areas showing different erosional and restoration/regeneration histories. The authors use biogeochemical observations to infer the history of the sites and relate these to the prokaryotic community observed. they argue that the inputs of carbon from fresh vegetation is key to controlling the community, not the water level and use the microbiological data to argue that microbiome could take long periods of time to recover from the impacts of erosion. The manuscript is clearly and concisely written, the combinations of techniques is appropriate to the study and their case is well argued.
I find myself, for the first time in my career, of having no major criticisms of a paper that has come across my desk to review. The manuscript has a handful of comments asking for clarifications of minor points or making observations of data that have not been discussed (perhaps because what looks like a obvious difference in a figure might not be statistically supported?)

Reviewer #3 (Remarks to the Author):

The manuscript entitled “Legacy of peatland erosion shapes microbial communities during recovery” aims to evaluate the composition of several elements, including DOC, CO2 amount, organic matter composition, C:N ratio, as well as microbial biomass and community composition across depth after peatland erosion and among three peatland degradation stages: degraded, restored, and naturally regenerated. The article is globally very well written, and the figures give valuable insight. The results are well described and sufficiently discussed in a proper manner.

However, I still have a few major and minor concerns, thus I would recomend to revise the article before publication.

Major concerns:

- Introduction: The introduction is clear and leads the reader to the subject. The global aim of the study is stated, but it is not

detailed enough and does not give the main research goal of this article. Moreover, no hypothesis in line with the literature is given by the authors. Thus, I suggest revising the last paragraph of the introduction to provide more details about the goals of this research and the hypotheses behind it.

- Material and methods: I thank the author for providing a lot of information regarding their sampling sites, their sampling methods, and the thorough description of the method used to assess several parameters. However, I have two main concerns here:

(1)The use of DNA concentrations as a proxy for biomass. First, quantification with a fluorescence assay is not sufficient to provide a real quantification of DNA concentration; for that, it is necessary to use a method such as qPCR or dPCR. Second, has this method been applied elsewhere? DNA is very useful to assess the community composition; however, as microorganisms can possess several copies of genes, DNA concentration does not reflect the number of cells present, thus I am questioning the possibility of using DNA as a proxy of biomass.

(2)The statistical analyses are not detailed enough. RDA is well described, but the authors also performed ANOVA, which is not explained within the methods. Why use both forward and backward selection? There is no statistical comparison for the different parameters tested across depth between the three peatland conditions. This could be a great improvement for the manuscript to add statistical analyses to compare these parameters using, for instance, linear mixed models.

Minor concerns:

L34-36: The sentence is a bit long and difficult to follow
L35: “upland bog being the”
L74-76: not clear, please consider rewriting this paragraph to improve clarity
L99: centre > center, the same elsewhere in the manuscript
L136: which > that
L142: “14,30, and 50 cm”
L174: centered
Figure 1: The lines that show the correspondence of the picture to the area are not visible enough; increasing their size could improve clarity
L292: had
Figure 6: “Non-metric multidimensional scaling (NMDS)”
L470: was made
L471: “and a thicker layer”
L472: “to host a disctinct”
Supplementary Figure A: centered

** Visit Nature Portfolio's author and referees' website at www.nature.com/authors for information about policies, services and author benefits**

Communications Earth & Environment is committed to improving transparency in authorship. As part of our efforts in this direction, we are now requesting that all authors identified as ‘corresponding author’ create and link their Open Researcher and Contributor Identifier (ORCID) with their account on the Manuscript Tracking System prior to acceptance. ORCID helps the scientific community achieve unambiguous attribution of all scholarly contributions. You can create and link your ORCID from the home page of the Manuscript Tracking System by clicking on ‘Modify my Springer Nature account’ and following the instructions in the link below. Please also inform all co-authors that they can add their ORCIDs to their accounts and that they must do so prior to acceptance.
https://www.springernature.com/gp/researchers/orcid/orcid-for-nature-research

For more information please visit http://www.springernature.com/orcid

If you experience problems in linking your ORCID, please contact the Platform Support Helpdesk.

Version 1:

Decision Letter:

** Please ensure you delete the link to your author home page in this e-mail if you wish to forward it to your coauthors **

Dear Dr Ring-Hrubesh,

Your manuscript titled "Legacy of peatland erosion shapes microbial communities during recovery" has now been seen by our reviewers, whose comments appear below. In light of their advice we are delighted to say that we are happy, in principle, to publish a suitably revised version in Communications Earth & Environment.

We therefore invite you to revise your paper one last time to address the remaining concerns of our reviewers. At the same time we ask that you edit your manuscript to comply with our format requirements and to maximise the accessibility and therefore the impact of your work.

EDITORIAL REQUESTS:

Please review our specific editorial comments and requests regarding your manuscript in the attached "Editorial Requests Table".

*****Please take care to match our formatting and policy requirements. We will check revised manuscript and return manuscripts that do not comply. Such requests will lead to delays. *****

Please outline your response to each request in the right hand column. Please upload the completed table with your manuscript files as a Related Manuscript file.

If you have any questions or concerns about any of our requests, please do not hesitate to contact me.

SUBMISSION INFORMATION:

In order to accept your paper, we require the files listed at the end of the Editorial Requests Table; the list of required files is also available at https://www.nature.com/documents/commsj-file-checklist.pdf .

OPEN ACCESS:

Communications Earth & Environment is a fully open access journal. Articles are made freely accessible on publication. For further information about article processing charges, open access funding, and advice and support from Nature Portfolio, please visit https://www.nature.com/commsenv/open-access

At acceptance, you will be provided with instructions for completing the open access licence agreement on behalf of all authors. This grants us the necessary permissions to publish your paper. Additionally, you will be asked to declare that all required third party permissions have been obtained, and to provide billing information in order to pay the article-processing charge (APC).

Please use the following link to submit the above items:
Link Redacted
** This url links to your confidential home page and associated information about manuscripts you may have submitted or be reviewing for us. If you wish to forward this email to co-authors, please delete the link to your homepage first **

We hope to hear from you within two weeks; please let us know if you need more time.

Best regards,

Somaparna Ghosh, PhD
Associate Editor,
Communications Earth & Environment
Consulting Editor,
Communications Sustainability

REVIEWERS' COMMENTS:

Reviewer #1 (Remarks to the Author):

After initial revisions, the authors did a very good job at incorporating and interpreting reviewer comments and criticisms. The addition of qPRC data strengthens this manuscript. I appreciate the author's commitment to address all concerns and suggested edits. I feel this version of the manuscript is written and presented well enough for publication.

Reviewer #2 (Remarks to the Author):

I have reviewed the re-submitted manuscript and am satisfied that the authors have addressed the concerns raised by the reviewers. The addition of the qPCR data to overcome questions raised about previous biomass estimates is most welcome. The only additional comment I would make is that the error bars on the qPCR data are very wide and so it is not clear

whether the differences seen in abundance of archaea and bacteria are statistically robust. However, since the authors have simply stated the findings and not put too much weight on this for the overall conclusions of the paper I think this is fine. Overall, it is my view that no further changes are required before acceptance.

Reviewer #3 (Remarks to the Author):

I greatly thank the authors for submitting this revised version of their manuscript. The authors have addressed all my concerns, and their work is now more impactful, precise, and reproducible. I notably appreciate the addition of qPCR to quantify the abundance of genes related to bacteria and archaea. I think this is a great addition. The authors revised their material well, which allows for more reproducibility. The results and the discussion are clear and bring a lot of interesting knowledge.

** Visit Nature Portfolio's author and referees' website at www.nature.com/authors for information about policies, services and author benefits**

Open Access This Peer Review File is licensed under a Creative Commons Attribution 4.0 International License, which permits use, sharing, adaptation, distribution and reproduction in any medium or format, as long as you give appropriate credit to the original author(s) and the source, provide a link to the Creative Commons license, and indicate if changes were made.
In cases where reviewers are anonymous, credit should be given to 'Anonymous Referee' and the source.
The images or other third party material in this Peer Review File are included in the article's Creative Commons license, unless indicated otherwise in a credit line to the material. If material is not included in the article's Creative Commons license and your intended use is not permitted by statutory regulation or exceeds the permitted use, you will need to obtain permission directly from the copyright holder.
To view a copy of this license, visit https://creativecommons.org/licenses/by/4.0/

The authors would like to thank all three Reviewers for the constructive points raised on our submitted manuscript. We have revised the manuscript to address the Reviewer's concerns, especially regarding the determination and interpretation of prokaryote abundance which was raised by both Reviewers 1 and 3, and about which a detailed note is provided below (Section 1). All other point-by-point responses to each comment are in Section 2 of this response, with the Reviewer's comments in black, our reply in red.
Thank you for your time and expertise.

1. Authors' note on the determination and interpretation of prokaryote abundance in the manuscript.

The authors thank reviewers 1 and 3 for raising related comments on the use of Qubit-derived DNA fluorescence as a proxy for microbial biomass in the original submitted manuscript. We accept that this assay is likely too crude to be confidently employed as a direct proxy for 'microbial biomass'. Instead, it reflects the potentially extractable double-stranded DNA in the sample, and is not specific to the prokaryotes which are of interest in this work.

To address the issues raised, we present new data derived by quantitative PCR (qPCR) analysis, to enumerate copy numbers of the 16S rRNA gene in the relevant samples. These qPCR reactions, were performed using primers specific to archaeal and bacterial 16S rRNA gene targets, employed as markers here due to presence in all prokaryote genomes. We used the DNA extracts carried out during the study which had been preserved at −70°C during the interim to ensure preservation.

We present gene copy numbers per gram of dry peat, an approach used in many previous published works on the peatland and wider soil microbiome. Such studies have presented qPCR analyses based on absolute quantification in the following terms; "Quantification of total bacterial and fungal microbial communities" (Barta *et al.* 2020); "Bacterial and archaeal population sizes were determined by quantifying 16S rRNA gene copies" (Kluber *et al.* 2020); "Quantitative PCR (qPCR) assays were used to quantify the total bacterial communities" (Allingham *et al.* 2024); "Bacterial and fungal abundance was determined through quantitative PCR"/"abundance of bacteria was estimated using the primers [...]" (Song *et al.* 2023).

The points regarding DNA extraction efficiency (Reviewer 1) and copy number differences between taxa (Reviewer 3), remain relevant to our updated method. As such, we adopt a more cautious approach to the presentation and interpretation of these results. Whilst we suggest in-text that they are likely to reflect relative differences in the underlying biomass of the two communities, we no longer characterise the results as a measure of the 'microbial biomass' throughout the text. Our qPCR results can instead be interpreted as an approximation of bacterial and archaeal abundance in the peat samples, with comparisons drawn between depths and between shallow peat under bare and regenerating surfaces.

qPCR methods are presented at lines 293-311. Results are referred to in section 3.3, including at lines 510-511; 538; 560; and 630.

2. **Point-by-point responses to all other reviewer comments:**

Text in black = reviewer comments   Text in red = authors response

Reviewer #1 (Remarks to the Author):

Review for Communications Earth & Environment Journal

The considered manuscript entitled "Legacy of peatland erosion shapes microbial communities during recovery" presents unique findings that expands our knowledge on how microorganisms adapt to differing ecological circumstances in a damaged peatland that is undergoing partial recovery. However, while I believe these results merits publication, some minor revisions should be made. I suggest minor revisions as my comments are editorial and do not require further experimentation or data generation.

Here are my line-by-line comments:

Line 20 - I recommend rewording this first sentence. It somewhat reads that "all peatlands are now C sources." We have revised the phrasing (line 20) to reflect our intended meaning:

"Human degradation has caused many peatlands worldwide to shift from long-term carbon sinks to net carbon sources"

Line 33 – In your first couple of paragraphs, I feel it is important to discuss vegetation types/cover. Are these eroded sites once Sphagnum dominated? What are the consequences of Sphagnum loss vs vascular plant loss (as well as loss of both)? Does vascular plant or Sphagnum moss contribute more to erosion and vis versa. I feel this is important to discuss as you mention many of these sites are ombrotrophic bogs. We have added mention of the particular importance of *Sphagnum* loss (line 52), due to its role in maintaining high water tables.

Line 41 – can establish We have revised the phrasing (line 54); where previously we used 'A positive feedback can become established' now we use 'A positive feedback may develop' to retain passive phrasing.

Line 42 – would "re-colonization" be more appropriate? Revised as suggested (line 55)

Line 43 – provide an appropriate citation 'Surface instability' was removed and clarified in detail, along with citations provided for the unfavourable conditions for vegetation re-establishment that can establish on bare peat. The conceptualisation as a 'positive feedback' is the authors own, but builds on the evidence provided in the previous

sentence (Vegetation loss -> enhanced erosion; with citations), and the current sentence (actively eroding peat -> impedes vegetation recovery). (Lines 53-56)

Line 77 – It would be good to describe what lead to the erosion scenarios in this peatland. You discussed the many ways this can occur in the previous paragraphs, but this specific circumstance should be made clear here. If it is not currently known, then that should be stated. This detail has been added in the site description section (L148-150):
"The eroded region was bare by the 1940s (earliest record is from aerial reconnaissance flights[38]), and though the exact cause of erosional onset is not known, a report on the condition of the site commissioned by the BBNPA[39] concludes that the most likely factors for the onset of erosion were grazing pressure and the deposition of atmospheric pollutants."

Line 77 & 114 – In reference to the area of managed restoration, this restoration practice should be briefly described, even while referencing another paper. This could be explained in a bit more detail in the methods as well. Additional description has been added immediately after first mention of the managed restoration site (L166-171):
"Management intervention consisted of blocking erosional gullies with heather bales and spreading heather brash across the adjacent bare peat surfaces. As of 2022, the bales had themselves become vegetated and trapped peat sediment, and would impede surface water flow such that pools form upslope after rainfall events."

Line 91 – I feel this methods section could use more detail. You have a dynamic system you're investigating, so providing the reader with as much information as possible is important here. What are the detailed differences between your three areas of the peatland complex? Additional detail added: Regarding the degraded peat area, information is now provided on the characteristics of the degraded surface which support our interpretation of 'active' erosion (lines 160-163). Regarding the managed restoration area, additional detail is given on intervention techniques, and the date of management intervention has been provided (lines 163-171). Regarding the natural regeneration area, the latest possible date of vegetation recolonisation has been included to support our characterisation of areas of long and short-term regeneration for this and the managed areas respectively (lines 159-160). In addition (as in original), there is information supplied on peat depths and vegetation types, though paragraph is reformatted for clarity to bring this information together while presenting the three areas under comparison in sequence (lines 151-184).

Line 114 – Do you have an idea as to why there is an area where natural restoration is occurring and not at the actively eroding area? We later (line 424-427) suggest that:

"At Waun Fignen Felen, exposure of peat layer with a higher bulk density and lower hydraulic conductivity (Supplementary Fig. C) may have facilitated a comparable transition in the region of natural regeneration."
We have added specific mention at this point of the natural regeneration area in this revision to add clarity.

Line 90 (methods) – Is there any data on hand, or recent studies that have surveyed any nearby peatlands that have not been affected or eroded? While I believe the study is good enough to stand on with the site selected, it would be very beneficial to have a relatively untouched reference peatland especially to compare vegetation communities. Regarding vegetation comparison with local sites, the authors have added a reference to vegetation community elsewhere in the Brecon Beacons by way of Chambers et al. 2013 who examined vegetation records from three profiles in the region (lines 174-177). All peatlands in the region exhibit signs of degradation however, so no untouched record can be provided. Regarding the general point on the study standing alone, the authors also note that we are not aware of any similar studies on microbial community composition in the region. Few metabarcoding records having been published from UK peatlands to date, stressing the novelty of this work.

Line 150-154 – looks like a different font or font size. Sentence updated from 11pt to 12pt (lines 213-217)

Line 218 – Can "wet chemistry methods" be explained in more detail? Details on how the FTIR bands were calibrated in the method paper cited (Hodgkins et al 2018) have been added (lines 278-281), providing the reader with relevant detail on the composition of the fractions. Previous reference to 'wet chemistry methods' is replaced by relevant techniques used: "Relevant bands were subsequently identified and processed using the method set out by Hodgkins et al.[43] to provide semi-quantitative %-aromatic and %-carbohydrate values. In that study, the aromatic fraction was calibrated against Klason lignin (acid-insoluble material minus ash), and the carbohydrate fraction against total cellulose + hemicellulose (determined by acid hydrolysis and HPLC following toluene and ethanol rinses)."

Line 232 – I would be very cautious about this interpretation. I do not agree that this can be used as a proxy for biomass. There is too much variability when extracting DNA from environmental samples, particularly peat (humic acids etc.). It would be safer to suggest that this is the amount of estimated/potentially extractable DNA from each sample or something of that nature. If you can find other studies that have used this as a proxy, that would help your argument but even then, I would be highly cautious with this. Reference to DNA quantification by Qubit fluorescence removed from methods (line

292), please see authors' response above regarding determination and interpretation of prokaryote abundance in the manuscript.

Line 248 – Be sure that every statistical application used in this study is described here. For example, you employ NMDS in your results but do not mention it here. We apologise for this oversight in the submitted manuscript. Additional description of NMDS as well as the ANOVA carried out on the redundancy analysis RDA, has been added (lines 365-371).

Line 322 – What kind of vegetation regrowth primarily? Sphagnum and/or vascular plants? Clarification added (line 444):
"All these observations are consistent with the contemporary regrowth of fresh vegetation (primarily mosses and graminoids) at the natural regeneration site."

Line 336 (Fig. 3 caption) – I would briefly explain how you calculated your fractional abundance. We have added detail on the processing methods for both the Py-GC-MS and FTIR data (line 465).

Line 374 – please see previous note on this interpretation of biomass. Line 508. Please see authors' response above regarding determination and interpretation of prokaryote abundance in the manuscript.

Line 398 – please see previous note on this interpretation of biomass. Line 532. Please see authors' response above regarding determination and interpretation of prokaryote abundance in the manuscript.

Line 417 (Fig. 5) – mentioning or showing where the water-table is situated (average or at time of sample collection) would be useful here. A personal opinion. Please provide n=?. While the authors agree that indicating the water table position would highlight that the apparent transition in the natural regeneration community is unrelated to water table position. The authors refrain from highlighting this position in the figure as it may instead lead readers to conclude that water-table is the most relevant parameter to consider when interpreting the figure.
n=X values have been added to the legend. (Line 563).

Line 500 – I feel your conclusion could be stronger. You already have a long Results and Discussion section explaining your data and your conclusion could bring forth the 'bigger picture' of your study. What does your data suggest about the ecological functioning of these sites, now and in the future? How are you linking microbial community changes and 'recovery?' We have reworked the conclusions section (line 664-674) to strengthen it, including by removal of text that repeated points already

made in previous sections of the manuscript. After a brief re-statement of the primary findings, we now make three points on the bigger picture: 1) Re. 'healthy' microbiome not recovering from bottom-up but top-down; 2) Re. potential timescales required for recovery, if as here recovery is associated with accumulation of new material at the surface; 3) Re. enhanced microbial processing of the renewed bioavailable C input, which is of relevance to those considering peatland C balance.

Reviewer #2 (Remarks to the Author):

This is a fine case study of a peatland with areas showing different erosional and restoration/regeneration histories. The authors use biogeochemical observations to infer the history of the sites and relate these to the prokaryotic community observed. they argue that the inputs of carbon from fresh vegetation is key to controlling the community, not the water level and use the microbiological data to argue that microbiome could take long periods of time to recover from the impacts of erosion. The manuscript is clearly and concisely written, the combinations of techniques is appropriate to the study and their case is well argued.
I find myself, for the first time in my career, of having no major criticisms of a paper that has come across my desk to review. The manuscript has a handful of comments asking for clarifications of minor points or making observations of data that have not been discussed (perhaps because what looks like a obvious difference in a figure might not be statistically supported?)
Comments submitted on attached PDF document:
L110: Earlier this site was described as having the benefit of "co-location of areas representing long- and short-term regeneration atop erosional surfaces, alongside ongoing surface erosion". Presumably these are listed in the same order but without an estimate on the date of natural revegetation, it is not possible to determine which of these sites is considered to be the "long-term regeneration site". Added clarification by describing the natural regeneration site (line 159-160):
"with aerial photographs confirming recolonisation by 1990 at the latest"

L117: The supplementary information references Fig 6 as having the locations, should that read Fig 1? The supplementary figure caption has been updated to reference Fig. 1

L285-287: This sentence isn't immediately clear - presumably what you mean is that there is evidence for historic surface peat loss, but two of the sites are currently accumulating peat? Sentence split in two (lines 400-403). Clarification added (additions are highlighted by underlining here):
"There was evidence for significant <u>historic</u> surface peat loss at all three study areas within the peatland, <u>with the degraded profile continuing to undergo active erosion</u>. <u>By contrast</u>, recent accumulation was visibly apparent in the top 3–5 cm of the restoration profiles and the upper 5–15 cm in the natural regeneration area."

L378: Is it just the 1.1c Thaumarchaeota that are reduced? What strikes me is the apparent disappearance of almost all the archaea in the naturally regenerated at the shallower depths We now include mention of archaeal abundance being lower in the natural regeneration profiles (at line 510-511) as suggested. This is supported by our

additional qPCR results: "Total archaeal abundance was lower throughout the natural regeneration depth profiles compared to the degraded and restoration areas (Fig. 5b)."

L395-410: The convergence of the communities is clear from the figure, but there also appears to be decrease in the proportion of the archaea discussed in this paragraph and at the same time an increase in the proportion of unclassified reads. does this say something about the unexplored diversity of natural restoration sites and about the roles for archaea at the different sites?

Re. the decreased proportion of archaea in the natural regeneration profile (which is further highlighted in our qPCR results) this is now mentioned in the text previously (lines 510-511). While in this paragraph (lines 535-551) we discuss depth trends in the abundance of different microbial groups. There is an increased contribution from the archaea, specifically *Thaumarchaeota* and *Bathyarchaeota*, which are mentioned, and their potential functional roles are discussed.

Re. an increase in proportion of unclassified reads, we understand this to refer to a greater proportion of the community represented by the 'Other' category in Fig. 5c. However, most reads under this 'Other' label were not unclassified, but rather are unlabelled in the figure to preserve legibility (justifiably due to low relative abundance in the community).

Reviewer #3 (Remarks to the Author):

The manuscript entitled “Legacy of peatland erosion shapes microbial communities during recovery” aims to evaluate the composition of several elements, including DOC, CO_2 amount, organic matter composition, C:N ratio, as well as microbial biomass and community composition across depth after peatland erosion and among three peatland degradation stages: degraded, restored, and naturally regenerated. The article is globally very well written, and the figures give valuable insight. The results are well described and sufficiently discussed in a proper manner.

However, I still have a few major and minor concerns, thus I would recommend to revise the article before publication.

Major concerns:

- Introduction:

The introduction is clear and leads the reader to the subject. The global aim of the study is stated, but it is not detailed enough and does not give the main research goal of this article. Moreover, no hypothesis in line with the literature is given by the authors. Thus, I suggest revising the last paragraph of the introduction to provide more details about the goals of this research and the hypotheses behind it.

After the context paragraphs, we have reworked the text of the penultimate paragraph (lines 85-95) that focuses on the specific knowledge gap, study aim, and principal hypothesis based on the literature. Specifically we have added the statements:

“The primary aim of this work is to determine the relative influence of substrate supply, bulk peat organic matter composition and water-table position on microbial community composition during peatland restoration.”

“Given the widely-reported controlling effect of water table position29, 35, we hypothesised that this would be a stronger predictor of microbial community composition during peatland recovery.”

We note that we do not finish the introduction on the aims and hypotheses due to the journal house style requirement to finish with a brief summary of findings, but we end by relating the principal finding back to the hypothesis above (lines 102-106).

- Material and methods:

I thank the author for providing a lot of information regarding their sampling sites, their sampling methods, and the thorough description of the method used to assess several parameters. However, I have two main concerns here:

(1)The use of DNA concentrations as a proxy for biomass. First, quantification with a fluorescence assay is not sufficient to provide a real quantification of DNA concentration; for that, it is necessary to use a method such as qPCR or dPCR. Second,

has this method been applied elsewhere? DNA is very useful to assess the community composition; however, as microorganisms can possess several copies of genes, DNA concentration does not reflect the number of cells present, thus I am questioning the possibility of using DNA as a proxy of biomass. Please see authors' detailed response above regarding changes to the determination and interpretation of prokaryote abundance in the manuscript.

(2)The statistical analyses are not detailed enough. RDA is well described, but the authors also performed ANOVA, which is not explained within the methods. Why use both forward and backward selection? **There is no statistical comparison for the different parameters tested across depth between the three peatland conditions. This could be a great improvement for the manuscript to add statistical analyses to compare these parameters using, for instance, linear mixed models.** Additional description of NMDS as well as the ANOVA carried out on the redundancy analysis RDA, has been added (lines 365-371). The authors apologise for this oversight in the initial submission.
Re. stepwise selection (lines 360-363), we have clarified the text on variable selection and include mention of our reasoning for stepwise regression which is to allow iterative re-evaluation of variable inclusion (method operates by forwards selection but also checking the previous term to see if it can now be eliminated, reducing sensitivity to early variable inclusion decisions).
We agreed with the reviewer that linear mixed models (LMMs) could support our interpretation of depth trends in the geochemical profiles in-text, and comparison of the degradation status. We have included this additional analysis in the resubmitted manuscript (methods at lines 334-345), with relevant LMM results referred to in-text (lines: 415-417; 438; 449; 471; 495-496; 489-491. In addition, a summary table of all LMM results is included in the Supplementary Information).

Minor concerns:

L34-36: The sentence is a bit long and difficult to follow Sentence has been split into two for clarity (lines 44-46)

L35: “upland bog being the” Revised as suggested (line 45)

L74-76: not clear, please consider rewriting this paragraph to improve clarity Paragraph has been reworked to read more clearly (lines 83-95), and focus on the interaction between water table and vegetation restoration. Originally a related but competing focus (regarding losses if C from modern vs long-term store) was introduced in the final sentence (~line 95, previously line 74-76), this has been removed.

L99: centre > center, the same elsewhere in the manuscript. Line 144. The authors have employed British English throughout the text and have therefore retained this usage for consistency. We are happy to alter this to suit the journal's preferred house style if required. As the Submission Guidelines and Style and Formatting Guide do not specify a requirement in this regard, we have maintained the existing usage for now.

L136: which > that Revised as suggested (line 198)

L142: "14,30, and 50 cm" We understand the comment to refer to removal of spaces in list (line 205). The spacing and punctuation in numerical lists have been retained as submitted for clarity, and can be adjusted during copy-editing if required: "6, 14, 30, and 50 cm"

L174: centered Line 237. See previous response regarding use of British English throughout

Figure 1: The lines that show the correspondence of the picture to the area are not visible enough; increasing their size could improve clarity The joining lines in the figure have been thickened to improve legibility (line 240).

L292: had Revised as suggested (line 408)

Figure 6: "Non-metric multidimensional scaling (NMDS)" Revised as suggested (Fig. 6, line 606)

L470: was made We have rephrased this sentence to address the unclear meaning in the previous iteration (line 625).

L471: "and a thicker layer" Revised as suggested (line 626)

L472: "to host a disctinct" Revised as suggested (line 627)

Supplementary Figure A: centered See previous response regarding use of British English throughout

The authors would like to thank the reviewers for their comments on the revised manuscript, and re-iterate their thanks for their reviews of the original manuscript that have led to an improved work. No further changes were required by the reviewers, though we have now made edits to satisfy the editorial requests that were made by the journal as set out below (and in the Cover Letter to the editors).

Point-by-point responses to reviewer comments

Reviewer #1 (Remarks to the Author): After initial revisions, the authors did a very good job at incorporating and interpreting reviewer comments and criticisms. The addition of qPRC data strengthens this manuscript. I appreciate the author's commitment to address all concerns and suggested edits. I feel this version of the manuscript is written and presented well enough for publication. Authors have made no further substantive changes in response to the reviewer comment

Reviewer #2 (Remarks to the Author): I have reviewed the re-submitted manuscript and am satisfied that the authors have addressed the concerns raised by the reviewers. The addition of the qPCR data to overcome questions raised about previous biomass estimates is most welcome. The only additional comment I would make is that the error bars on the qPCR data are very wide and so it is not clear whether the differences seen in abundance of archaea and bacteria are statistically robust. However, since the authors have simply stated the findings and not put too much weight on this for the overall conclusions of the paper I think this is fine. Overall, it is my view that no further changes are required before acceptance. Authors have made no further substantive changes in response to the reviewer comment

Reviewer #3 (Remarks to the Author): I greatly thank the authors for submitting this revised version of their manuscript. The authors have addressed all my concerns, and their work is now more impactful, precise, and reproducible. I notably appreciate the addition of qPCR to quantify the abundance of genes related to bacteria and archaea. I think this is a great addition. The authors revised their material well, which allows for more reproducibility. The results and the discussion are clear and bring a lot of interesting knowledge. Authors have made no further substantive changes in response to the reviewer comment

Note on changes to manuscript since latest reviewer comments

To suit the journal style as set out in the Editorial Requests Table, the authors have moved the Methods section to the end of the manuscript main text. To facilitate reader understanding of the Results and Discussion section, a brief statement on the study design and the ‘Site overview and sampling scheme’ figure (Figure 1 in all

versions) have been relocated from the Methods to the start of the Results and Discussion.

The statement added to the Results and Discussion at line 100 consists of the following text:

This study examined three hydrologically independent areas within the region of severe historic erosion at Waun Fignen Felen which each represent contrasting states of recovery (Figure 1). These were 1) a 'degraded' area which was actively eroding with a predominantly bare surface; 2) an area of 'managed restoration' which has become re-vegetated following intervention to block adjacent gullies in 2009; and 3) an area of 'natural regeneration' in which vegetation has naturally recolonised a former erosional surface, with aerial photographs confirming recolonisation by 1990 at the latest (detailed site information can be found in the Methods section).

The following text was correspondingly removed from the Methods at line 389:

We examined three areas of the peatland, each deemed to be hydrologically independent,

And the following removed at line 390:

Our three sampling sites consisted of: 1) a 'degraded' area which was actively eroding with a predominantly bare surface; 2) an area of 'managed restoration' which has become re-vegetated following intervention to block adjacent gullies in 2009; and 3) an area of 'natural regeneration' in which vegetation naturally recolonised a former erosional surface, with aerial photographs confirming recolonisation by 1990 at the latest.

In addition, minor edits have been made to suit the journal style (as set out in the Editorial requests table; e.g. p-value exact values reported where appropriate, reference to Fig. have been changed to Figure throughout). No other substantive changes have been made.

Legacy of peatland erosion shapes microbial communities during recovery

Fin Ring-Hrubesh[1,2*], Mike Vreeken[1,2,3], Anne Eberle[1,‡], Bradley Welch[4], Paul Sinnadurai[4], Penny Johnes[2,5], Robert Griffiths[6], Angela Gallego-Sala[7], Richard Pancost[1,2,3], Casey Bryce[1,2*]

1 School of Earth Sciences, University of Bristol, Wills Memorial Building, Queens Road, Bristol BS8 1RJ, UK

2 Cabot Institute for the Environment, Royal Fort House, University of Bristol, BS8 1UH

3 Organic Geochemistry Unit, School of Chemistry, University of Bristol, Cantock's Close, Bristol, BS8 1TS, UK

4 Bannau Brycheiniog National Park Authority, Brecon, Powys, LD3 7HP, UK

5 School of Geographical Sciences, University of Bristol, University Road, Bristol BS8 1SS, UK

6 School of Environmental and Natural Sciences, Bangor University, Bangor, Gwynedd, LL57 2UW, UK

7 Geography, College of Life and Environmental Sciences, University of Exeter, Amory Building, Rennes Drive, Exeter, EX4 4RJ, UK

‡ Present address: GFZ Helmholtz Centre for Geosciences, Telegrafenberg, 14473 Potsdam, Germany

*** Corresponding authors: Fin Ring-Hrubesh (fin.ring-hrubesh@bristol.ac.uk) and Casey Bryce (casey.bryce@bristol.ac.uk)**

Abstract

Human degradation of peatlands worldwide has turned them into net carbon sources. In upland blanket peatlands, erosion disrupts new plant-derived carbon input and exposes deep peat, putting old carbon at risk of oxidation.

The efficacy of restoration in preventing carbon loss and recovering ecosystem function depends on microbial responses to both water table manipulation and renewed litter input. Yet it is unclear how these factors alter the microbial communities that ultimately control carbon storage and emissions.

We show that microbial community composition in the eroded peatland of Waun Fignen Felen, South Wales, was primarily governed by the bioavailability of organic matter rather than water-table position. Long-term erosion leaves behind a legacy of highly degraded organic matter, unaltered by re-wetting. Where plant litter accumulation is renewed on formerly eroded peat surfaces, the influx of bioavailable organic input supports a distinct microbial community with greater biomass, and evidence of elevated respiration.

1. Introduction

Northern peatlands operate as a large terrestrial carbon store (400–600 GtC; [1-3]), and in the UK peatlands cover 8–12% of the terrestrial surface with ombrotrophic upland bog the dominant peatland type (65% of peatland area)[4]. Peatland surface erosion and vegetation loss are common facets of upland blanket bog degradation[5]. Vegetation loss can be driven by several factors including excessive grazing pressure[6], die-off due to intolerance of atmospheric pollutant and nutrient deposition[7, 8], and fire[9, 10]. As vegetation acts to stabilise the peatland surface, its disappearance can lead to enhanced erosion by surface water flow, wind action and frost-heave[11-13]. A positive feedback can become established where surface instability hinders the re-establishment of vegetation, such that bare peat surfaces persist along with associated carbon losses.

Peatland carbon can be lost as both a direct and indirect result of erosional processes. Organic matter is washed out from the peat profile directly, entering watercourses as particulate organic matter with detrimental consequences for water quality[14], and further decomposing in streams to dissolved organic carbon (DOC) and CO_2[15]. In severely eroded UK blanket peatlands, Pawson *et al.*[16] observed that carbon losses through fluvial export alone total 29–106 MgC km^{-2} yr^{-1}. Stored carbon is also destabilised as an indirect consequence of erosion, where gully formation drives water table drawdown and enhanced mineralisation in the deepened oxic layer[17]. Peatland drying leads to increased CO_2 emissions and a suppression of CH_4 emissions with a net warming effect overall[18, 19]. Although impacts of drying on DOC production and export are reported, there is greater uncertainty over the direction of the response[20-22], with DOC representing a heterogeneous pool of microbial and plant metabolites governed by a wide range of biotic and abiotic processes. To address both direct and indirect carbon loss pathways, restoration of eroded peatlands will typically aim to stabilise peat surfaces and re-establish vegetation, while also implementing re-wetting measures.

Typically, the composition of peatland organic matter follows a depth gradient, with deep peat dominated by less reactive organic compounds, such as lignin and polyphenols, which have persisted over time[23]. While the anaerobic decomposition of these molecular classes does occur, it proceeds slowly, leading to their relative persistence[24]. Conversely, recently deposited near-surface peat will contain more 'labile' organics such as polysaccharides which are typically more microbially available[25]. In a system where surface peat has been lost, the

contrast in the composition of shallow and deep organic matter could be lessened or even absent[26].

The impact of altering the physico-chemical environment (by concurrent re-wetting and vegetation re-establishment) on microbial communities is uncertain, yet the microbiome response is of major relevance given its central role in the peatland carbon cycle and ecosystem function[27, 28]. While reduced microbial activity has been reported in more mineralised peats in several studies[26, 29, 30], it is unclear to what extent peat macromolecular composition limits carbon degradation in practice, given the generally overriding effect of water table position. Decoupling these drivers is particularly relevant for peatland restoration where water table and vegetation recovery may be superimposed onto a degraded peat matrix. In these contexts, the degree to which peatland microbes can utilise and release modern carbon or carbon from the long-term store is key to the function of the ecosystem as either a carbon sink or source.

We carried out an examination of microbial communities at the upland ombrotrophic bog of Waun Fignen Felen in South Wales. The site serves as a case study, due to the co-location of areas representing long- and short-term regeneration atop erosional surfaces, alongside ongoing surface erosion. We present the geochemical and microbial characterisation of regenerating and degraded peat profiles with the aim of determining the strongest predictors of microbial community composition. We show that peat bulk organic matter composition differed most strongly across depth profiles where peat was actively accumulating. Where long-term recovery had occurred, this surface layer supported a microbial community distinct from that in degraded surface peats exposed by erosion, and separate to the deep peat microbiome. We find that during recovery from erosion, the switch to supply of recent substrate input from vegetation, superimposed onto otherwise low-bioavailability bulk peat can exert an even stronger influence on microbial community composition than water table position.

2. Materials & methods

2.1 Site overview

The study site, Waun Fignen Felen, is an ombrotrophic bog situated on a limestone escarpment south of Fan Hir, South Wales (475 m AOD; 51.8465, –3.7103). The peat stratigraphy in the region of the peatland under investigation was characterised by Smith and Cloutman[31], who describe *Sphagnum* spp. peat forming a raised mound overlying

Phragmites spp. peat deposited during earlier reed-swamp conditions atop late Devensian lake sediments. The region is differentiated from the blanket peat in the surrounding landscape by more severe erosion, greater peat depth and by radial drainage outwards from its centre. In their study, Smith and Cloutman[31] reported conventional radiocarbon dates for five near-surface (<10 cm) samples from across this eroded region, of between 4850 ±70 BP and 3800 ±70 BP (uncalibrated[31, 32]), indicating that erosion had exposed ancient peat.

The presence of ongoing erosion alongside areas representing short- and long-term regeneration within the same peatland complex was a key factor in site selection. As such, we can ascribe the primary differences in geochemistry to the degree of recovery from severe erosion. We examined three areas of the peatland, each deemed to be hydrologically independent, and all situated within the eroded region identified by Smith and Cloutman[31]. Our three sampling sites consisted of: 1) an actively eroding area with a predominantly bare surface; 2) an area which has become re-vegetated following management intervention to block adjacent gullies in 2009; and 3) an area in which vegetation has naturally recolonised a former erosional surface. In the naturally recolonised profile, an abrupt transition between amorphous, dark peat and an upper layer predominantly composed of identifiable plant material marked the inferred former erosional horizon.

In the area of managed restoration, the dominant plants were *Deschampsia flexuosa*, *Hypnum jutlandicum* and *Campylopus introflexus*. In the naturally regenerating area, *Eriophorum angustifolium*, *Sphagnum* species (*capillifolium*, *fimbriatum*, *palustre*) and occasional stands of *Molinia cerulea* were the primary vegetation cover (Supplementary Fig. A). Measured peat depths in the degraded and restoration areas were 3.8–4.3 m, compared to 1.2–2.2 m in the area of natural-regeneration. The shallower peat in the latter area resulting from its position away from the centre of the peatland (basal dates from Smith and Cloutman[31] did not indicate a younger age) and may have been further diminished by a greater extent of historic peat loss in this area which was examined further in this study.

2.2 Water table monitoring and porewater sampling

In each study area water table position was monitored in a centrally located dip-well, while porewater was sampled from three porewater membrane-exchange equilibrator (PWE) samplers each installed 2 m apart. Water table data was collected using pressure transducers calibrated against ambient barometric pressure at the site (HOBO U20L-01). The dip-wells

were not anchored to underlying geology, and any vertical shift was recorded and manually re-set on a 6-weekly basis, with a linear correction applied to the intervening data when shifts were observed. As such, water level depths are reported relative to the peat surface. Summary rainfall data at the site (daily totals) were recorded using a ‘tipping-bucket’-type gauge (Ecowitt WH5360).

PWE samplers for the collection of filtered porewater were built according to the specification of Bowes and Hornibrook[33], itself modelled on a sampler design proposed by Hesslein[34]. These samplers consisted of a PVC housing which seals a series of horizontal cells behind a polyether sulfone (PES) membrane (0.2 µm pore size, Pall Life Sciences HT-200). The cells were then connected on either side to tubing, which was routed to the surface while the samplers were installed within the peat. Tubing for water sample collection was connected to the cell base, while the top of each cell was connected to N_2-filled syringes during sampling to maintain an anoxic headspace. After sampling, the wells were refilled with N_2-sparged de-ionised water. Samples were collected from depths of 6, 14, 30 and 50 cm at 6-week intervals between August 2021–October 2022, allowing sufficient time for equilibration between porewater and PWE wells between each sampling event. The samples collected were filtered with 0.2 µm PES syringe filters into N_2-flushed vials and transferred to cold storage at 5°C within 12 hours prior to analysis.

2.3 Porewater geochemical analyses

Porewater pH was recorded within 12 hours using a calibrated probe (Thermo Scientific Orion ROSS Ultra). DOC concentrations were determined within 72 hours using the non-purgeable organic carbon (NPOC) method (Shimadzu TOC-L). Select dissolved inorganic ions were determined by spectrophotometric methods (NH_4^+-N, total NO_3^--N + NO_2^--N, PO_4^{3-}-P, SO_4^{2-}-S using Thermo Scientific Discrete Industrial Analyzer System Reagents for Water/Environmental Samples; total dissolved Fe by the ferrozine method), with only those selected for inclusion in the RDA model presented below. Dissolved CO_2 and CH_4 were determined during 4 of the sampling events (2022: 19th May, 6th July, 26th August, 11th October) using the widely applied headspace-equilibration method[35]. Briefly, immediately after the anoxic sampling of porewater from the equilibrator samplers into a syringe, an equal volume of N_2 gas was drawn from a gas-tight bag before the sample was shaken vigorously with the headspace to equilibrate for 60 seconds. Headspace was then transferred into a vacuum vial (Exetainer) and stored at room temperature until analysis. Concentrations in the headspace samples were determined using gas chromatography (Agilent 7890A), equipped with a flame ionisation

detector (FID; for CH_4, and CO_2 after methanisation to CH_4). Dissolved concentrations in the original porewater were determined by application of Henry's Law using water temperatures recorded by the dipwell loggers, with Henry's Law constants and temperature dependence coefficients sourced from the NIST database[36].

2.4 Peat sampling

Peat was sampled in summer 2022 (27th June–1st July), with cores taken using a D-section (Russian type) corer. Within each of the three study areas, a nested sampling strategy was employed with 5 peat sampling locations spaced 20 m apart along a 'W'-shape transect, the central position of which was co-located with the dipwell and porewater sampling (Fig. 1). In the restoration area, each sampling location was sited 2 m away from a blocked gully. Sampling at each position was conducted in triplicate, and all peat was transferred onto dry ice in the field prior to freezer storage at −20°C within 12 hours. Additionally, cores were subsampled in the field using sterile tools to retrieve 4 cm long subsamples, at depths centred on 6, 14, 50, 105 cm, and the peat base, which were transferred into centrifuge tubes for DNA analysis and stored at −80°C on return to the laboratory.

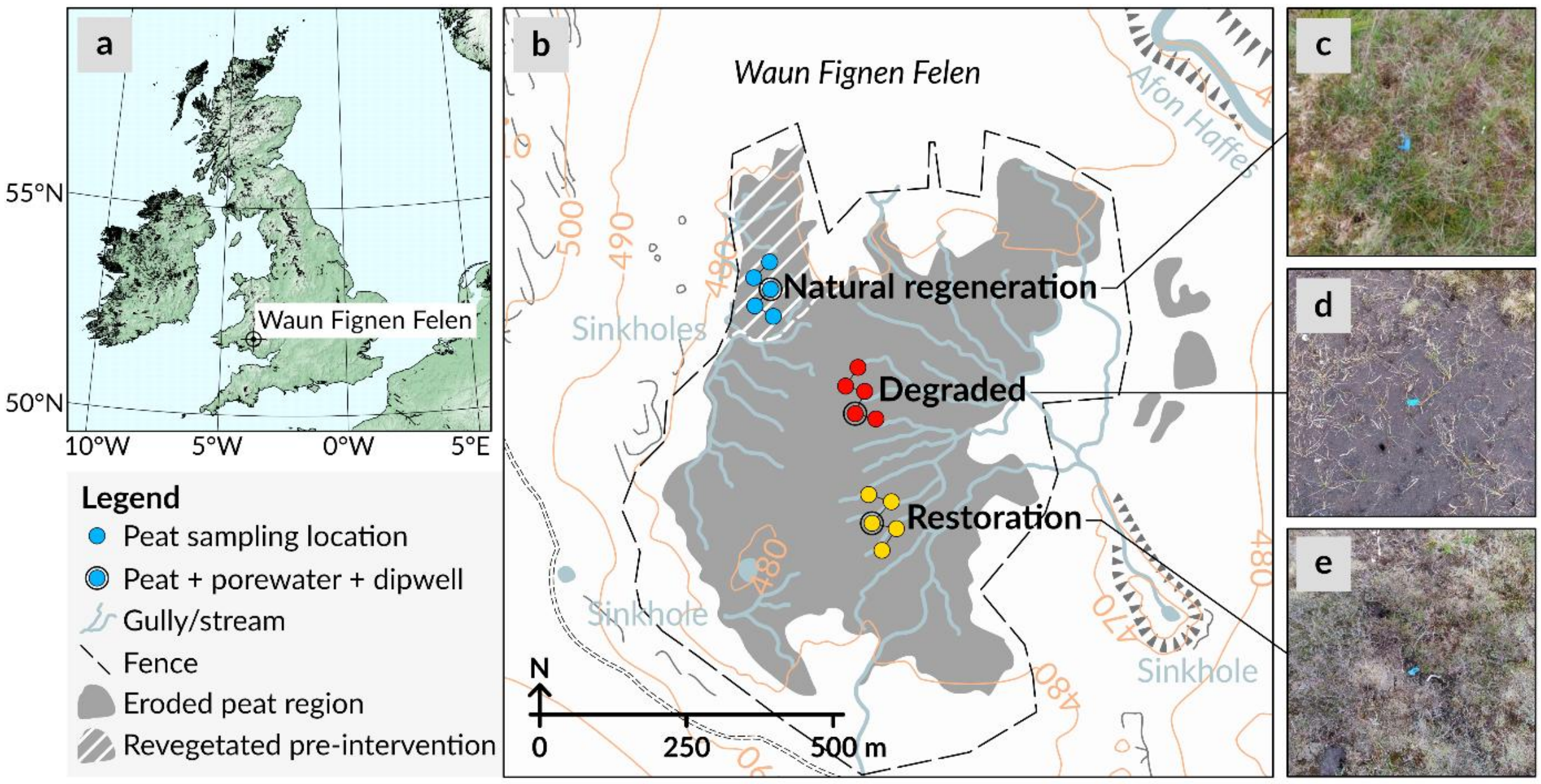

Figure 1 | Site overview and sampling scheme. a Location of Waun Fignen Felen in the British Isles, peatland regions are indicated by black shading[37]. **b** Locations of dipwells, peat and porewater sampling within the fenced study region at Waun Fignen Felen. **c–e** Photographs of the peat surface at the sampling locations; each image covers approximately 1 x 1 m of the ground surface.

2.5 Peat geochemical analyses

The composition of organic macromolecules in the bulk peat was investigated using pyrolysis-gas chromatography-mass spectrometry (Py-GC-MS), at the same depths at which DNA sequencing was carried out. Triplicate samples were analysed, from the central 3 positions in each region (Fig. 1). For this analysis, 0.5±0.1 mg of peat was taken from the freeze-dried, and homogenised subsamples. Peat in furnaced quartz tubes was subjected to an initial thermal desorption step at 300°C for 2 minutes, to remove free lipids that may obscure the lignocellulosic signal. The desorbed peat was subsequently pyrolysed at 610°C for 20 seconds using a Chemical Data System 6200 series pyroprobe with the trap at 50°C. The pyrolysates were released post-pyrolysis by heating to 300°C for 4 minutes, and were transferred at 310°C to a gas chromatograph (GC; Thermo Scientific Trace 1310) fitted with an Rtx-1 column (dimethyl polysiloxane, non-polar, 60 m × 0.32 mm ID, film 0.25 µm). The GC oven was set to heat from 40°C (held initially for 4 min) to 300°C at 40°C min^{-1}, with helium used as the carrier gas. The GC was coupled with a single quadrupole mass spectrometer (Thermo Scientific ISQ7000), with the detector scanning from *m/z* 50-650. Peak identification was carried out in XCalibur (Thermo Scientific) using inhouse libraries and literature. The relative abundances of components were determined from the peak areas from integrated extracted ion chromatograms of pyrolysis product major ions as in Schellekens *et al.*[38], and are reported as a proportion of the sum of all those identified. The compound classes of Schellekens *et al.*[38] were also used to group products, with additional components classed according to Chen *et al.*[39] (2,5-Dimethylfuran) and Nowakowski *et al.*[40] (Desaspidiniol). These fractional abundances enable comparison between the relative abundances of compound classes, which is inherently qualitative[41]. Within the polysaccharides, levoglucosan was used to represent the relative abundance of cellulose as it is the primary pyrolysis product[42]; all other polysaccharide pyrolysis products were grouped to represent the hemicellulose fraction. Lignin was represented functionally through phenol, guaiacol and syringol pyrolysates, while other compound classes identified were the aliphatics (alkane/alkene doublets), N-containing compounds, and aromatics. These additional compound classes affect the relative abundances of the discussed classes, but not the ratios between them.

Fourier-transform infrared spectrometry (FTIR) was conducted complementarily on the same samples to analyse the quality of the peat organic matter. FTIR spectra were obtained by averaging 16 scans of the 4000 to 600 cm^{-1} region (4 cm^{-1} resolution, 1 cm^{-1} data interval; PerkinElmer Spectrum Two). Freeze-dried and homogenized samples were placed on an ATR crystal with pressure applied to attain even coverage and contact. An automatic background correction was applied to all samples using the instrument software. Relevant bands were subsequently identified and processed using the method set out by Hodgkins *et al.*[43] to provide relative abundances for aromatic and carbohydrate fractions, which they calibrated using wet chemistry methods to provide semi-quantitative %-aromatic and %-carbohydrate values. Additionally, the total carbon and nitrogen content of the pyrolysis/FTIR subsamples was determined using an elemental analyser calibrated with reference standard NC Soil 341506 (EA; Elementar Vario MACRO Cube), with C:N ratios computed using these mass fractions. Cores taken at the porewater sampling locations were subsampled at 10 cm intervals for determination of peat bulk density profiles by drying a known volume of sample at 105°C.

2.6 DNA extraction and sequencing

Peat subsamples for DNA extraction were stored at –80°C and were handled in a laminar flow hood, with a homogenised sample extracted across the whole 4 cm depth interval by scouring a groove along the frozen sample with a fine spatula. This scoured peat material (*ca.* 500 mg) was processed using DNeasy PowerSoil Pro extractions kits (QIAGEN). Extract concentrations were quantified using a fluorescence assay (ThermoFisher Qubit 2), and DNA concentrations were corrected for mass of raw peat to provide total soil microbial DNA, used here as a proxy for biomass.

PCR amplification, library preparation and sequencing were carried out commercially by Novogene (Cambridge, UK). The V4 region of the 16S rRNA gene was targeted using the Earth Microbiome Project (EMP)[44] primer pair 515F (5'-GTGCCAGCMGCCGCGGTAA-3'); 806R (5'-GGACTACHVGGGTWTCTAAT-3'), before PCR products of the correct size were selected by gel electrophoresis. These primers were selected as they were designed as universal primers to target both bacteria and archaea and have been widely applied[45]. An equal amount of PCR products was pooled from each sample and end-repaired, A-tailed and further ligated with Illumina adapters. Libraries were then sequenced on an Illumina MiSeq platform to generate 250 bp paired-end raw reads.

After sequencing, the DADA2 algorithm[46] was used to distinguish amplicon sequence variants (ASVs) and their relative abundance within each sample, using standard settings for trimming and quality filtering. Assignment of taxonomy was carried out using the SILVA database (v.138.1)[47]. ASVs which were assigned to chloroplasts and mitochondria were removed, and the sequence tables were randomly rarefied to an even size using the phyloseq R package[48].

2.7 Statistical analysis

To determine the key environmental controls on the microbial community, redundancy analysis (RDA) was applied across the depths for which highly replicated sequencing data, as well as porewater samples were retrieved (6, 14, 50 cm). Porewater (annual mean) and peat geochemical parameters were used as explanatory variables while greenhouse gases were excluded as they were considered equally likely to be response variables. To avoid overrepresentation of multivariate geochemistry datasets, dimensionality reduction was carried out whereby the summary ratios of polysaccharides : lignin and hemicellulose : cellulose were used to represent the bulk organic geochemistry (as determined by Py-GC-MS), rather than each compound or compound class; while ammonium and oxides of nitrogen were grouped as 'dissolved inorganic N'. To determine which combination of variables explained the most variance in microbial community composition (ASV counts), step-wise removal of the remaining variables took place according to their multicollinearity. This was assessed by the VIF scores (Variance Inflation Factors) of each variable when used as RDA constraints, and redundant variables were removed until those remaining had low VIF scores ($VIF < 5$)[49]. Subsequently, forward and backward stepwise selection of variables was performed using the ordistep function in the vegan R package[50], whereby iterative addition and removal of variables was conducted based on Akaike's Information Criterion (AIC). The final RDA thus retains only those variables that significantly contributed to the explained variation in the ASV abundance data and is plotted with type 2 scaling to show the effect of explanatory variables.

3. Results & Discussion

3.1 Water table regimes align with regeneration status

Over the two-year monitoring period, water tables were consistently highest in the natural regeneration site, intermediate in the managed restoration site and lowest in the degraded site (Fig. 2). This trend, aligning with the degradation status, was magnified by more rapid drops in water table in the degraded and restoration areas during periods of low precipitation (Supplementary Fig. B). None of the site water tables were extremely deep, with mean water table in the degraded peat at 12.9 ±10.1 cm below the surface, compared to 9.7 ±8.9 cm in the restoration profile, and 5.5 ±7.2 cm in the naturally regenerating area (± standard deviations). Across the monitoring period Waun Fignen Felen received >2400 mm of annual rainfall. This is considerably above the minimum typically associated with upland blanket peatland (1600 mm[51]). The high water tables across the whole site are likely supported by this high annual precipitation as well as the relatively flat topography of the eroded site, conferring lower rates of runoff.

3.2 Extent of recovery evident in geochemical profiles

There was evidence for significant surface peat loss at all three study areas within the peatland, with recent accumulation visibly apparent in the top 3–5 cm of the restoration profiles and the upper 5–15 cm in the natural regeneration area. Despite this, the bulk density of surface peat was comparable across the three conditions. The shallower peat base in the natural regeneration site was evident in the bulk density profiles (rapid increase associated with the peat base; Fig. 2), and elevated bulk density values were seen in this profile between 40–110 cm relative to the degraded and restoration areas.

Natural regeneration was associated with a surface layer that has the highest C:N ratio. In the degraded and restoration areas, lower C:N ratios at 6, 14 and 30 cm corresponded with the absent or thin accumulating layers in these profiles, suggesting that shallow peat in these areas is more decomposed than in the natural regeneration region, as differential loss of C during decomposition has been reported widely[52, 53]. Beneath the natural regeneration surface layer, there was a transition to amorphous peat, which was reflected in the steep decline in C:N ratios in this profile compared to consistent values downcore in the other areas (Fig. 2).

While the peat profile in the naturally regenerating area may have been somewhat shallower due to its location further from the centre of the raised peat dome, the differences in peat physical properties also indicate that prior erosion may have been more advanced in this area, having exposed older deep peat before the onset of natural recovery. Regeneration atop erosional horizons, has been described in other UK peatlands [54, 55], with Milner *et al.*[55] setting

out the case whereby exposure of an erosion-resistant layer allows the system to transition to a new stable state in which renewed peat accumulation can proceed. At Waun Fignen Felen, exposure of peat layer with a higher bulk density and lower hydraulic conductivity (Supplementary Fig. C) may have facilitated a comparable transition.

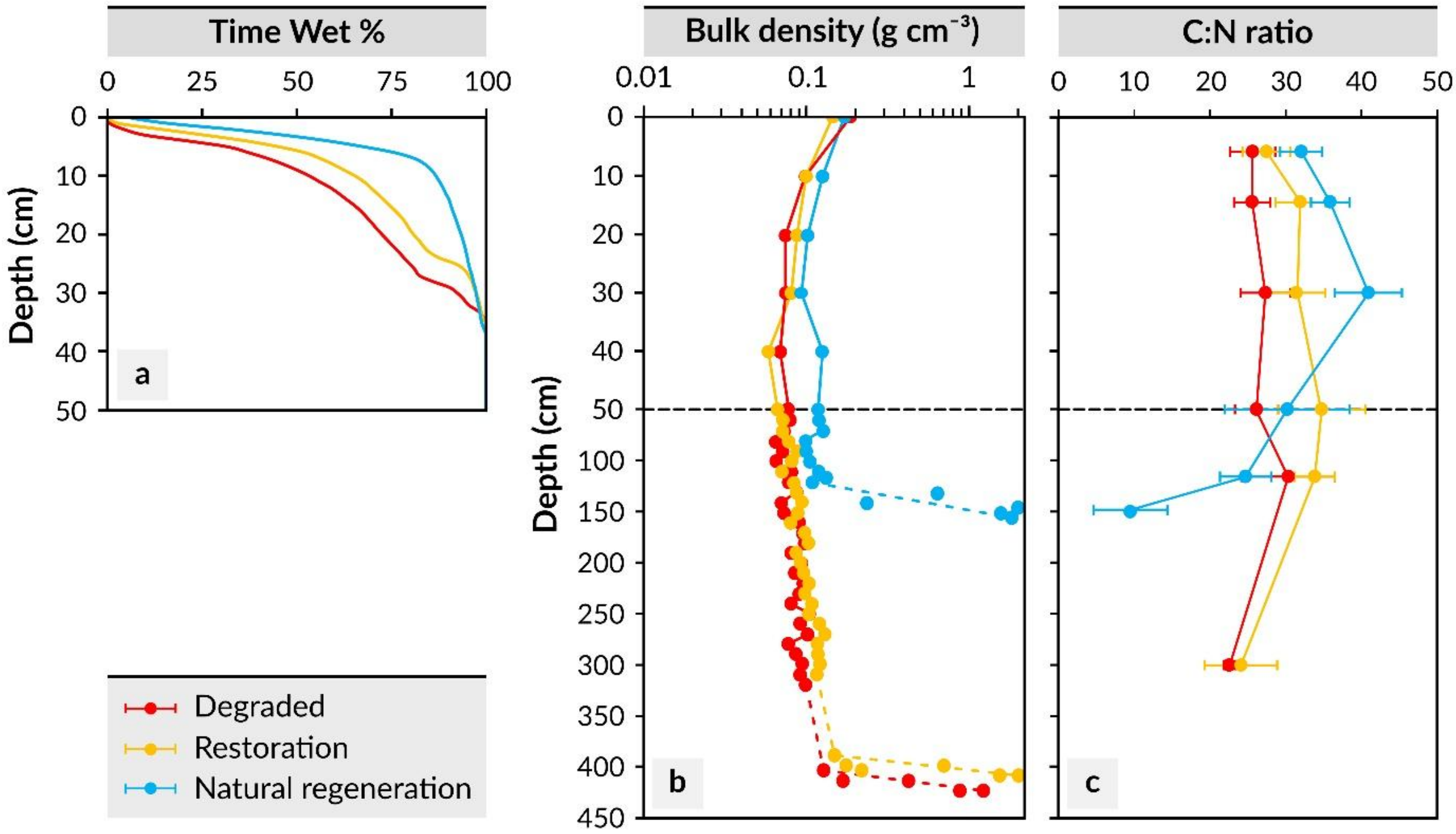

Figure 2 | Summary of peat properties across the three conditions. a The proportion of time for which peat depths were below the water table during the two week period prior to core sampling, below 50 cm peat was permanently inundated during the entire monitoring period. **b** The peat bulk density (dry mass/*in situ* volume) at 10 cm intervals, presented along a log-scale due to steep change at peat base. Values were derived from the core taken at the porewater sampling location. A solid line is used to connect datapoints; near the peat base a dashed line is used to approximate the trend. **c** The carbon-to-nitrogen ratio of bulk peat across all cores, with error bars representing the standard deviation. For depths 6, 14, and 50 cm n=15 (3 replicate cores at 5 locations); for depths 30, 105 and 147/300 cm n=3 (3 locations). Note change in y-axis scale at 50 cm.

Peat macromolecular composition, as assessed using Py-GC-MS and FTIR, also reflected the recovery status, driven by a combination of historical factors, including burial depth and erosion (Fig. 3). In shallow samples the central role of fresh plant-derived input was apparent, with a greater proportion of labile, polysaccharide-derived compounds present in the vegetated areas. Most polysaccharides decompose preferentially to lignin, such that all of the peat in the study area, except that in the shallow peat of re-vegetated sites, has become polysaccharide-depleted; this occurs throughout the depth profile at the degraded site where fresh vegetation is absent. In addition, the polysaccharide pool within shallow samples from

vegetated profiles contained a greater relative contribution from hemicellulose-derived pyrolysis products than cellulose (Fig. 3), indicating fresh accumulation of plant material. By contrast, markers of lignin and other phenolics (and aromatic signals in FTIR, representing approximately the same pool) were depleted in the shallow peat, especially at the two revegetated sites. All these observations are consistent with the contemporary regrowth of fresh vegetation at the natural regeneration site.

With increased depth, the proportion of pyrolysates associated with lignin, as well as the aromatic FTIR signal, increased. This is expected with downcore degradation of peat organic matter, but we suggest that this trend primarily reflects the switch from fresh vegetation to more strongly decomposed peat below the erosion horizon. This would explain the lack of a downcore change at the degraded bare surface sites, where vegetation recovery is absent or limited. The increase in lignin/aromatic components with depth at the natural regeneration sites was particularly steep, so much so that their proportions were greater than at the degraded and restored sites (similar to C:N profiles). Given that all three sampling locations were sited in the basin set out by Smith and Cloutman[31], with comparable dates of peat initiation; the shallower peat depth now present in the natural regeneration area; and greater inferred erosional loss; we suggest that the high aromatic content reflects older peat exposed in that area, potentially with different, more lignin-rich vegetation inputs (greater contribution from vascular plants with depth; for example in Biester *et al.*[56]).

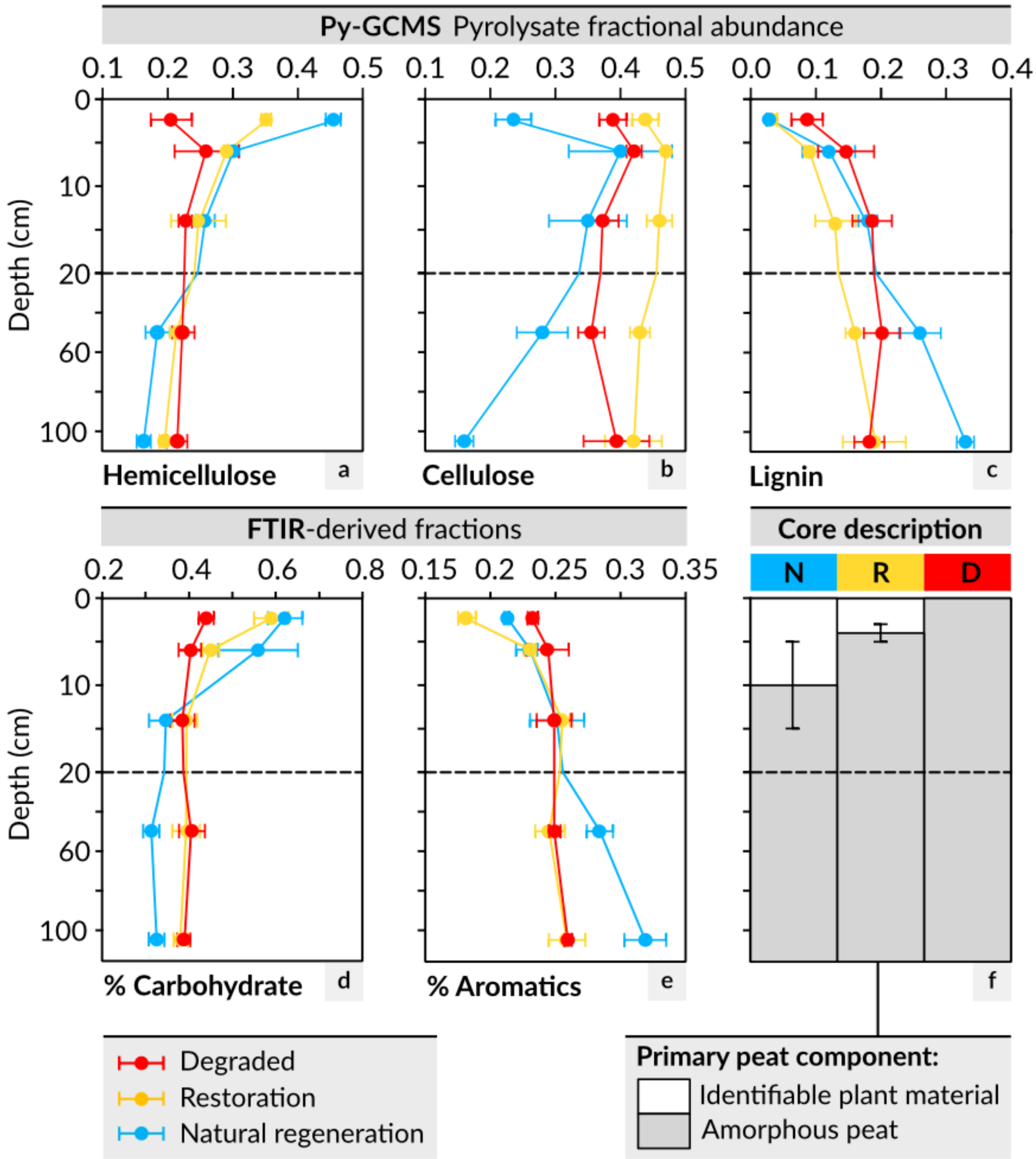

Figure 3 | Peat organic matter composition. A comparison of relative abundances of organic macromolecular groups in bulk peat as derived by Py-GC-MS (**a**–**c**) and FTIR (**d**–**e**) from triplicate samples collected in summer 2022. Note change in y-axis scale at 20 cm. Error bars for Py-GC-MS and FTIR-derived fractions represent the standard deviation between samples. **f** An overview of the position of the erosional horizon within profiles, with error bars indicating the observed range in position.

Porewater concentrations of dissolved organic carbon (DOC) were notably lower in the degraded area than either of the re-vegetated areas (Fig. 4). Studies on DOC release after peatland restoration report highly contrasting outcomes, and when reviewing available data Evans *et al.*[20] found only limited support for any decrease associated with restoration, and Darusman *et al.*[57] show no consistent effect. In cases such as ours where apparent DOC

production is instead lower in degraded sites than under recovery, several explanations have been suggested: Comparing eroded and intact peatland sites in the Pennines, Evans *et al.*[58] attributed lower DOC export from eroded sites to either lower 'lability' or minimal interaction between water and the peat matrix. Elsewhere a recovery of plant productivity has been reported to cause increased porewater DOC[59], while Pinsonneault *et al.*[60] demonstrate higher water extraction of DOC from fresh *Sphagnum* and vascular plant material than from litter or peat material. Bernard-Jannin *et al.*[22] report that DOC export did not decrease under restoration, instead being sustained by a supply of more recent organic matter at the peat surface. We found that where highly aromatic macropolymers comprised a greater proportion of the organic substrate, this was associated with lower accumulation of dissolved organic compounds in porewater. In the naturally regenerating area, where porewater DOC was highest, there was a temporally consistent peak at a depth of 14–20 cm (Fig. 4). The peak in DOC concentrations in the near-surface, and association between higher concentrations and areas with vegetated peat surfaces, suggest that it originates from modern plant matter through litter or root exudates. This would represent a more accessible pool of potential substrates for microbial metabolism than DOC derived from the bulk peat organic matter[60].

Correspondingly, dissolved CO_2 was elevated in the region of natural regeneration throughout the downcore profile, despite the higher water table in this profile, whereas CO_2 concentrations were low under enhanced degradation, pointing to the lower availability of the remaining organic matter. Our interpretation that elevated CO_2 under the re-vegetated scenario predominantly results from the cycling of modern plant-derived carbon, would imply that this is not detrimental to the long-term carbon store. Yet this would need to be confirmed by further investigation of the peatland carbon balance, particularly as priming of microbial communities by labile carbon input has been proposed to enhance peat decomposition[61-63]. Our combined data suggest that re-vegetation may act as an overriding control on CO_2 production, with restoration adding labile inputs that are superimposed onto low bioavailability bulk peat in formerly-eroded peatlands. No similar effect was observed with regard to methane concentrations, which were similar between the conditions (Supplementary Fig. D).

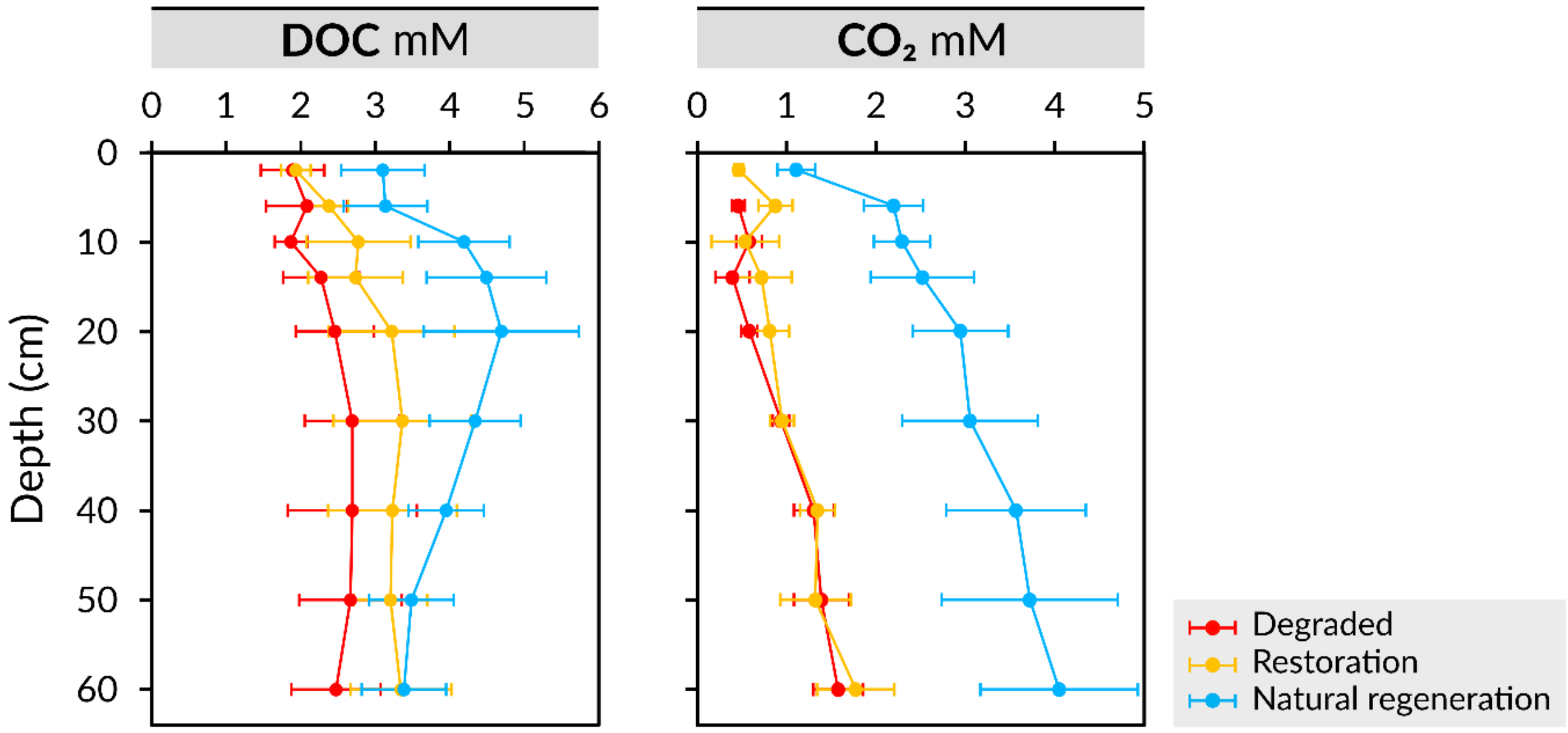

Figure 4 | Depth profiles of dissolved organic carbon and carbon dioxide in porewaters at Waun Fignen Felen. Points represent mean values with standard deviation shown by bars. Dissolved organic carbon was sampled on 12 occasions between August 2021–October 2022, CO_2 samples were collected on 4 occasions between May–October 2022.

3.3 Microbial community composition is vertically stratified

In the upper part of the profiles (6 and 14 cm), the natural regeneration area hosted more microbial biomass, as assessed by extractable DNA, than either the bare or restoration peat profiles (Fig. 5). In shallow peat, the microbial community composition in the natural regeneration locations was distinct from that in the degraded and restored areas (Fig. 5), with a markedly greater abundance of members of the order *Acidobacteriales* and family *Xanthobacteraceae*, and lower abundances of Group 1.1c *Thaumarchaeota* and the *Sideroxydans* and *Spirochaeta*.

Acidobacterial sequences are frequently among the most abundant in peatlands, with *Acidobacteriales* one of two orders which are dominant in acidic bogs globally (alongside *Bryobacterales*, which were less abundant here)[28, 64, 65]. A dominance of *Acidobacteriales* in the surface litter layer and subsequent decline in deeper peat has also been reported by Golovchenko *et al.*[66] in pine swamp peatland. The order includes both aerobic and anaerobic acidophilic chemoorganoheterotrophs, known to degrade a wide range of mono-, di-, and polysaccharides (Dedysh and Oren, 2020). The other taxon which was more abundant in the natural regeneration area, was the *Xanthobacteraceae*, elsewhere found to be common among the acrotelm microbiome[66-68]. These are chemoorganoheterotrophs with some strains capable

of sulfur oxidation and others able to fix nitrogen[69]. By contrast, the near-surface of the managed-restoration and degraded areas was enriched in the genera *Sideroxydans* and *Candidatus Nitrosotalea* (Fig. 5), both of which are known chemolithotrophs. These differences in the community composition, as well as apparent biomass, correspond with the finding of greater resource-availability in the naturally regenerating area due to a greater abundance of labile organics relative to the peat everywhere else at the site.

The composition of the microbial community began to converge in samples retrieved from below 50 cm and clustered closely in deeper samples, where peat composition and conditions were similar across the three conditions (Fig. 6). This was accompanied by a decline in apparent biomass in all the sites, relative to the upper profile, with similar low yields of microbial DNA in all three locations below 50 cm (<0.2 ng/mg peat; Fig. 5). The deep-peat community was enriched in members of the genus *Spirochaeta*, which contains obligate and facultative anaerobes involved in saccharolytic fermentation[70]. Also more abundant were the *Thaumarchaeota*, of which members described to date have been chemolithoautotrophic with some additionally capable of utilising simple organics heterotrophically[71]. In the deepest samples, proximal to the peat base, an increase in *Bathyarchaeota* abundance was observed. Archaea from this phylum could be key degraders of organic matter in peatlands particularly at depth, given their similar dominance in deep marine cores[72] and the diverse strategies for carbon assimilation that have been attributed to them, ranging from lignin metabolism to methanogenic CO_2 uptake[73, 74]. These findings support the inference that the lower rates of heterotrophic microbial activity observed in deep and highly decomposed peats[30, 75, 76], reflect the lower bioavailability of remaining substrate.

Many of the taxa associated with deep peat also dominated in the shallow degraded and restoration sites, and as a result the vertical change in community abundance was weaker in those profiles (Fig. 5). At these depths, peat was permanently inundated at all locations, although the organic matter composition diverged among them (Fig. 3), primarily due to the relatively elevated content of aromatic polymeric material at depth in the natural regeneration profile.

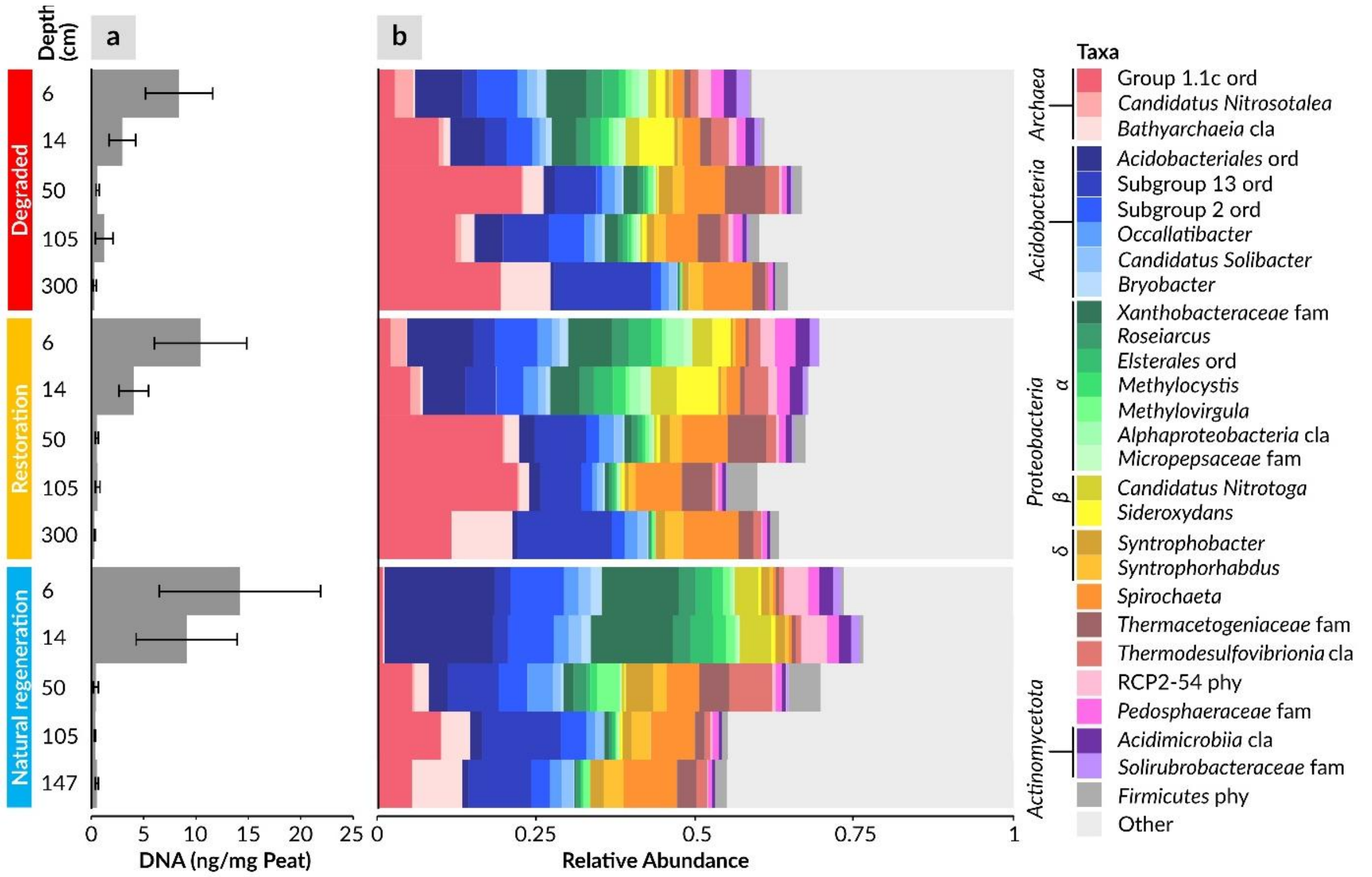

Figure 5 | a Microbial biomass across depths at Waun Fignen Felen. Extracted DNA per g of peat is used as a proxy for biomass, error bars represent standard deviation between samples. **b Microbial community composition.** Mean relative abundance of the most abundant 28 taxa across all samples with taxa ordered by phylum. Where identification at the genus-level was not possible the rank presented is indicated by abbreviation.

3.4 Organic matter quality shapes microbial community structure

To assess the relevance of the geochemical parameters measured in this study on the observed community composition, redundancy analysis (RDA) was employed after selection of the strongest explanatory variables as detailed previously. To assess the role of water table position, the proportion of time for which each sampling depth was below the measured water level ('time wet') was calculated over the preceding 2 weeks. Water level position was not parameterised directly as this is both highly intercorrelated with other depth-related parameters (including lignin proportion, dissolved ammonium and pH), and is less closely related to oxygen availability, the mechanism by which wetting is primarily expected to impact microbes. There was greater difference for time wet among the three sites than was derived from the dip to water level.

The RDA (Fig. 6a) reflected the convergence of the community at depth, with the greatest spread in relation to factor 1. The contrasting communities in the shallow peat were also represented in the RDA with minimal overlap, primarily explained by factor 2. This

clustering was the same as that present in non-constrained ordination using NMDS (non-metric multidimensional scaling; Fig. 6b), indicating that the environmental factors included could account for much of this difference. After sequential removal of explanatory variables, the 6 primary environmental factors included in the redundancy analysis (RDA) were the hemicellulose : cellulose ratio, dissolved inorganic N content, lignin : polysaccharide ratio, DOC concentration, pH and proportion of time below the water table (Fig. 6a). The proportion of time below the water table, accounted for less variance than either of the variables relating to peat organic geochemistry. The shallow community under natural regeneration clustered separately along the RDA2 axis, associated most strongly with differences in the hemicellulose : cellulose ratio and DOC concentration. At 50 cm the communities of the three states clustered more closely and were differentiated from the shallow peat community by a spread along the RDA1 axis. This depth-related spread was strongly associated with dissolved inorganic N and the ratio of lignin : polysaccharides. Depth trends in dissolved inorganic N across the site were predominantly derived from elevated ammonium and not oxidised N species (nitrate and nitrite; Supplementary Fig. E) at depth. As a result of the strong depth trend, dissolved inorganic N was the strongest predictor of variance identified (Table 1), but was of limited relevance to the treatment (between-state) effect observed in shallow peat. In unconstrained ordination (NMDS), clustering according to degradation history was also observed, and where the same environmental factors are fitted, a comparable relationship with community composition can be seen (Fig. 6b).

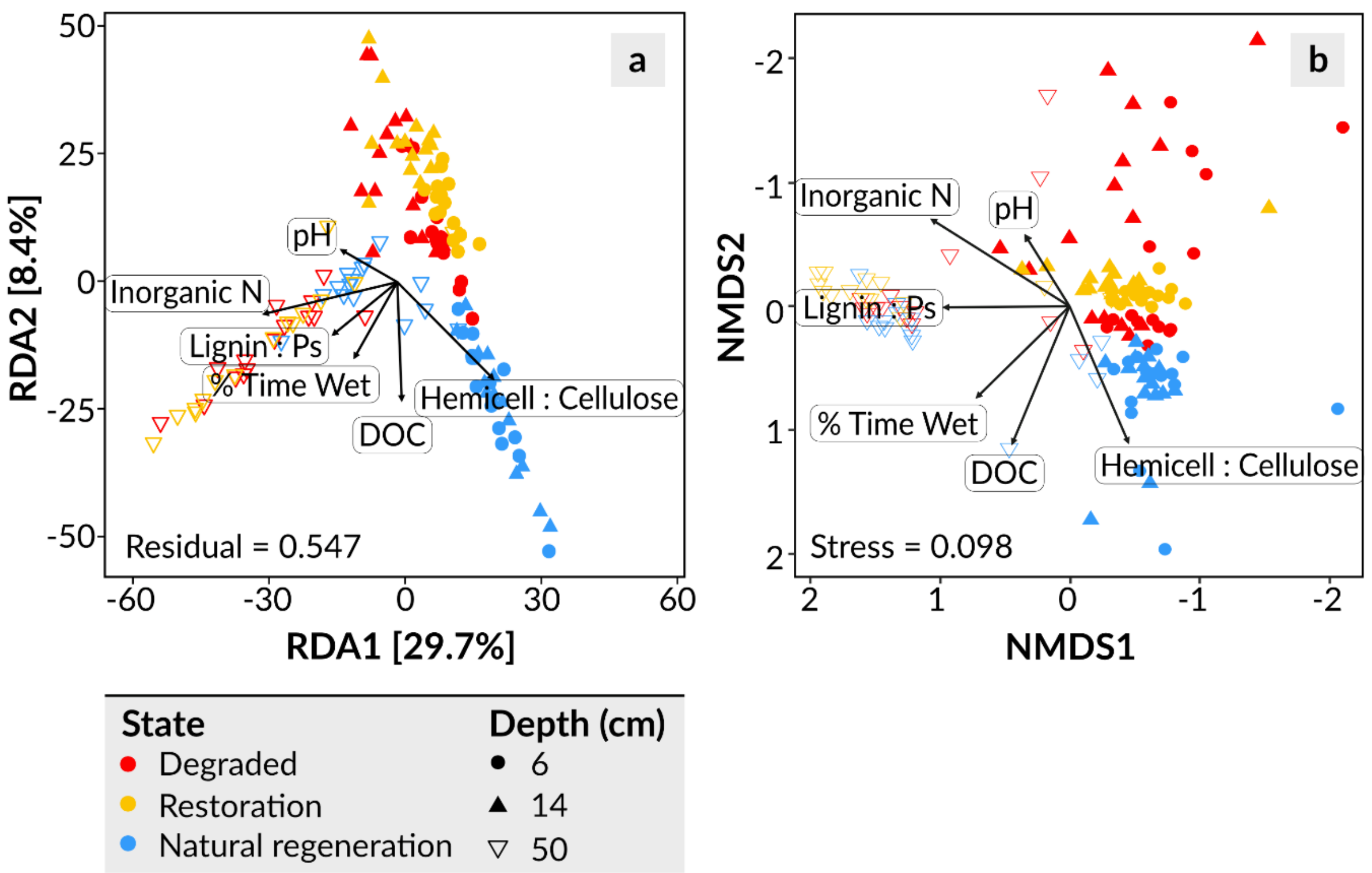

Figure 6 | Geochemical drivers of the microbial community. a Redundancy analysis (RDA) with geochemical parameters that contributed significantly to the explained variation in the ASV abundance.
b Non-metric multidimensional scaling based on distance matrices computed from ASV abundances using the Bray-Curtis method, the same environmental variables selected for the RDA are fitted to the unconstrained ordination using the envfit function in the vegan R package.

Parameter	Variance (%)	F-statistic	p-Value
Dissolved inorganic N	12.6	23.7	0.001
Hemicellulose:Cellulose	8.1	15.2	0.001
Lignin:Polysaccharide	4.1	7.6	0.002
DOC	3.8	7.2	0.001
pH	1.8	3.3	0.021
Time wet %	1.7	3.2	0.014

Table 1 | Results of ANOVA from redundancy analysis (RDA). Assessment of variables which significantly influenced community composition. All parameters are statistically significant at the $p<0.05$ threshold.

The time that peat layers were below the water table did not emerge as a strong driver (Table 1), although this might have been expected given the dominant role of water table in regulating oxygen availability and thus microbial processes[19, 77, 78]. Here, the depth trends in the composition of the microbial community were largely decoupled from the water table differences. If water table was to act as the primary control, the more frequent and deeper drying of the upper profiles in the degraded and restoration areas would be expected to

support a stronger vertical gradient in prokaryote community composition. Instead, the natural-regeneration profiles displayed a more marked contrast between the communities of shallow and deep peat despite more stable inundation throughout the profile, with the shallow to deep contrast instead corresponding to the boundary of accumulating and old peat.

Redundancy analysis indicated that in shallow peat, differences between the community composition (spread along RDA2; Fig. 6a), were most strongly explained by the hemicellulose : cellulose ratio, an indicator of a higher supply of relatively fresh plant substrate. Elsewhere the enzyme activity of peatland decomposer communities has been shown to be more sensitive to the quality of surface litter than to water table drawdown[79]. Here we found that community composition followed a similar trend, though in our case, the comparison was between an absence of litter in the degraded area, a thin layer of vegetation under recent restoration (3–5 cm) and thicker layer (5–15 cm) in the area of natural regeneration. Other studies have similarly found bare peat to host to a distinct community relative to long-term vegetated comparisons, and to support lower levels of microbial biomass and activity[80, 81]. We suggest that the higher lability of surface material may sustain the higher microbial biomass observed in the upper profile under natural regeneration, while CO_2 concentrations of more than double those observed in the degraded and restoration areas were another indication of greater microbial respiration. Again, microbial growth in the degraded peat is apparently restricted by factors other than water table position, differences in which were small at the site.

Past mineralisation likely caused the enhanced relative stability of the deep and erosionally exposed peat, with the residual organic matter being less reactive due to the greater proportion of aromatic compounds. Lower respiration and decomposition rates[26, 30, 75], enzyme activity[76], reduced microbial biomass[29, 82] have been attributed to poor substrate quality in more highly decomposed peats, due to a higher aromatic content. Here we find the tight clustering of the deep peat microbiome (Fig. 6) to be strongly aligned with geochemical parameters (dissolved inorganic N content, and lignin : polysaccharide ratio), rather than the water table. Furthermore, in those profiles without or with only a thin regenerated layer, dissolved CO_2 was lower than observed under natural regeneration, which may reflect a lower basal rate of respiration where the peat substrate is of limited availability. Although we examined dissolved CO_2 concentration and not emission, such findings have consequences for the stability and continued release of peat carbon after restoration, and therefore our interpretation of restoration success. They suggest that in systems where past carbon loss was

extensive while water tables remain high, the rate of microbial carbon utilisation would be limited as a consequence of altered organic matter quality. It should be noted that this is not evidence of a barrier to future degradation, or a resilience of peatlands to complete remineralisation, as proposed priming effects[61-63], or more extreme drainage than in the peatland studied here could still have the potential to lift the constraints on degradation of otherwise 'recalcitrant' compounds[83].

4. Conclusions

This study investigated differential recovery on former erosional horizons, a widespread feature of UK upland peatlands, by examining the site at Waun Fignen Felen, South Wales. We aimed to identify differences in the composition and availability of peat substrates and determine key drivers of the microbial community composition. We were particularly interested in the competing roles of water table position and vegetation recovery where both processes have occurred in an eroded setting. In this study, differences in water table position were consistent with their degradation history, yet play only a limited role in shaping the microbiome. Instead, the renewal of plant litter input at the peat surface was the predominant control on microbial community composition, with communities in the recovering layer expected to utilise this more bioavailable recent organic matter. Given the stratification of the microbial community with fresh peat accumulation, longer-term recovery (decadal timescales in this study) or transplant of plant communities may be required for microbiome shifts to occur on formerly eroded peat.

Acknowledgements

This work was funded by a Natural Environment Research Council (NERC) PhD studentship at FRESH Centre for Doctoral Training in Freshwater Biosciences and Sustainability (GW4+ FRESH CDT) awarded to F. Ring-Hrubesh [NE/R011524/1]. C. Bryce and F. Ring-Hrubesh are grateful for support from a Royal Society Research Grant [RGS/R2/202221] and the University of Bristol/Research England Policy Support Fund. M. Vreeken and A. Eberle were funded through PhD studentships at the Great Western Alliance Doctoral Training Partnership (NERC GW4+ DTP2) [NE/S007504/1]. We thank NERC for partial funding of the National Environmental Isotope Facility (NEIF; NE/V003917/1) and the project Climate, Energy and

Carbon in Ancient Earth Systems, awarded to R. D. Pancost, selected by the ERC and funded by UKRI [EP/X023214/1].

The authors thank the technical staff in the Schools of Earth Sciences and Geography at the University of Bristol (Fotis Sgouridis, Harry Li-Kam-Tin, Monica Huerta-Lopez, Ioanna Petropoulou); and the wardens of the Bannau Brycheiniog National Park Authority who supported the research fieldwork (Steffan Edwards, Wyn Morgan).

Author contributions

Fin Ring-Hrubesh: conceptualization, methodology, investigation, data curation, formal analysis, visualization, writing – original draft preparation.

Casey Bryce: conceptualization, funding acquisition, methodology, investigation, data analysis, supervision, writing – original draft preparation

Mike Vreeken: methodology, data curation, formal analysis, investigation, writing – review & editing

Anne Eberle: conceptualization, methodology, investigation, writing – review & editing

Rich Pancost: conceptualization, funding acquisition, methodology, data analysis, supervision, writing – review & editing

Angela Gallego-Sala: conceptualization, funding acquisition, methodology, supervision, writing – review & editing

Robert Griffiths: conceptualization, funding acquisition, methodology, data analysis, supervision, writing – review & editing

Bradley Welch: conceptualization, funding acquisition, investigation, supervision, writing – review & editing

Penny Johnes: conceptualization, funding acquisition, methodology, writing – review & editing

Paul Sinnadurai: conceptualization, writing – review & editing

All authors contributed substantially to the article and approved the submitted version.

Competing interests

The authors declare no competing interests.

Data availability

All data displayed in figures 2–5a, and raw geochemical datasets are available at Zenodo (https://doi.org/10.5281/zenodo.17368979). Raw sequencing data have been deposited at NCBI in the Sequence Read Archive (SRA) under BioProject accession number PRJNA1346434 (https://www.ncbi.nlm.nih.gov/bioproject/PRJNA1346434).

References

1. Yu, Z., Loisel, J., Brosseau, D.P., Beilman, D.W., and Hunt, S.J. Global peatland dynamics since the Last Glacial Maximum. *Geophysical Research Letters*. **37**:L13402 (2010).
2. Yu, Z.C. Northern peatland carbon stocks and dynamics: a review. *Biogeosciences*. **9**:4071-4085 (2012).
3. Hugelius, G., *et al.* Large stocks of peatland carbon and nitrogen are vulnerable to permafrost thaw. *Proceedings of the National Academy of Sciences*. **117**:20438-20446 (2020).
4. Evans, C., *et al.* Implementation of an Emissions Inventory for UK Peatlands. *Report to the Department for Business, Energy and Industrial Strategy, Centre for Ecology and Hydrology, Bangor*. (2017).
5. Evans, M. and Warburton, J. Fluvial Processes and Peat Erosion in *Geomorphology of Upland Peat* (eds., M. Evans and J. Warburton) 76-103 (Royal Geographical Society, 2007).
6. Parry, L.E., Holden, J., and Chapman, P.J. Restoration of blanket peatlands. *Journal of Environmental Management*. **133**:193-205 (2014).
7. Smart, S.M., *et al.* Impacts of pollution and climate change on ombrotrophic Sphagnum species in the UK: analysis of uncertainties in two empirical niche models. *Climate Research*. **45**:163-176 (2010).
8. Larmola, T., *et al.* Vegetation feedbacks of nutrient addition lead to a weaker carbon sink in an ombrotrophic bog. *Global Change Biology*. **19**:3729-3739 (2013).
9. Yeloff, D.E., Labadz, J., and Hunt, C. Causes of degradation and erosion of a blanket mire in the southern Pennines, UK. *Mires and Peat*. **1**:04 (2006).
10. Worrall, F., *et al.* Carbon fluxes from eroding peatlands – the carbon benefit of revegetation following wildfire. *Earth Surface Processes and Landforms*. **36**:1487-1498 (2011).
11. Shuttleworth, E.L., Evans, M.G., Hutchinson, S.M., and Rothwell, J.J. Peatland restoration: controls on sediment production and reductions in carbon and pollutant export. *Earth Surface Processes and Landforms*. **40**:459-472 (2015).
12. Foulds, S.A. and Warburton, J. Significance of wind-driven rain (wind-splash) in the erosion of blanket peat. *Geomorphology*. **83**:183-192 (2007).
13. Groeneveld, E.V.G. and Rochefort, L. Polytrichum Strictum as a Solution to Frost Heaving in Disturbed Ecosystems: A Case Study with Milled Peatlands. *Restoration Ecology*. **13**:74-82 (2005).
14. Aspray, K.L., Holden, J., Ledger, M.E., Mainstone, C.P., and Brown, L.E. Organic sediment pulses impact rivers across multiple levels of ecological organization. *Ecohydrology*. **10**:e1855 (2017).

15. Goulsbra, C.S., Evans, M.G., and Allott, T.E.H. Rates of CO_2 efflux and changes in DOC concentration resulting from the addition of POC to the fluvial system in peatlands. *Aquatic Sciences*. **78**:477-489 (2016).
16. Pawson, R.R., Evans, M.G., and Allott, T.E.H.A. Fluvial carbon flux from headwater peatland streams: significance of particulate carbon flux. *Earth Surface Processes and Landforms*. **37**:1203-1212 (2012).
17. Evans, M. and Lindsay, J. The impact of gully erosion on carbon sequestration in blanket peatlands. *Climate Research*. **45**:31-41 (2010).
18. Huang, Y*., et al.* Tradeoff of CO_2 and CH_4 emissions from global peatlands under water-table drawdown. *Nature Climate Change*. **11**:618-622 (2021).
19. Evans, C.D*., et al.* Overriding water table control on managed peatland greenhouse gas emissions. *Nature*. **593**:548-552 (2021).
20. Evans, C.D., Renou-Wilson, F., and Strack, M. The role of waterborne carbon in the greenhouse gas balance of drained and re-wetted peatlands. *Aquatic Sciences*. **78**:573-590 (2016).
21. Ritson, J.P*., et al.* The effect of drought on dissolved organic carbon (DOC) release from peatland soil and vegetation sources. *Biogeosciences*. **14**:2891-2902 (2017).
22. Bernard-Jannin, L*., et al.* Hydrological control of dissolved organic carbon dynamics in a rehabilitated Sphagnum-dominated peatland: a water-table based modelling approach. *Hydrology and Earth System Sciences*. **22**:4907-4920 (2018).
23. Tfaily, M.M*., et al.* Vertical Stratification of Peat Pore Water Dissolved Organic Matter Composition in a Peat Bog in Northern Minnesota. *Journal of Geophysical Research: Biogeosciences*. **123**:479-494 (2018).
24. Verbeke, B.A*., et al.* Latitude, Elevation, and Mean Annual Temperature Predict Peat Organic Matter Chemistry at a Global Scale. *Global Biogeochemical Cycles*. **36**:e2021GB007057 (2022).
25. Artz, R.R.E., Chapman, S.J., and Campbell, C.D. Substrate utilisation profiles of microbial communities in peat are depth dependent and correlate with whole soil FTIR profiles. *Soil Biology and Biochemistry*. **38**:2958-2962 (2006).
26. Leifeld, J., Steffens, M., and Galego-Sala, A. Sensitivity of peatland carbon loss to organic matter quality. *Geophysical Research Letters*. **39**:14704 (2012).
27. Robinson, C.H*., et al.* Aspects of microbial communities in peatland carbon cycling under changing climate and land use pressures. *Mires and Peat*. **29: 2** (2023).
28. Andersen, R., Chapman, S.J., and Artz, R.R.E. Microbial communities in natural and disturbed peatlands: A review. *Soil Biology and Biochemistry*. **57**:979-994 (2013).
29. Könönen, M*., et al.* Deforested and drained tropical peatland sites show poorer peat substrate quality and lower microbial biomass and activity than unmanaged swamp forest. *Soil Biology and Biochemistry*. **123**:229-241 (2018).
30. Schimmel, H., Braun, M., Subke, J.-A., Amelung, W., and Bol, R. Carbon stability in a Scottish lowland raised bog: potential legacy effects of historical land use and implications for global change. *Soil Biology and Biochemistry*. **154**:108124 (2021).
31. Smith, A.G. and Cloutman, E.W. Reconstruction of Holocene vegetation history in three dimensions at Waun-Fignen-Felen, an upland site in South Wales. *Philosophical Transactions of the Royal Society of London. B, Biological Sciences*. **322**:159-219 (1988).
32. Dresser, Q. University College Cardiff radiocarbon dates I. *Radiocarbon*. **27** (1985).
33. Bowes, H.L. and Hornibrook, E.R.C. Emission of highly ^{13}C-depleted methane from an upland blanket mire. *Geophysical Research Letters*. **33**:04401 (2006).
34. Hesslein, R.H. An in situ sampler for close interval pore water studies1. *Limnology and Oceanography*. **21**:912-914 (1976).
35. Kling, G.W., Kipphut, G.W., and Miller, M.C. The flux of CO_2 and CH_4 from lakes and rivers in arctic Alaska. *Hydrobiologia*. **240**:23-36 (1992).
36. NIST, NIST chemistry web book. 2023, National Institute of Standards and Technology, Gaithersburg, USA.
37. Xu, J., Morris, P.J., Liu, J., and Holden, J. PEATMAP: Refining estimates of global peatland distribution based on a meta-analysis. University of Leeds. [Dataset]. (2017).

38. Schellekens, J., *et al.* Influence of source vegetation and redox conditions on lignin-based decomposition proxies in graminoid-dominated ombrotrophic peat (Penido Vello, NW Spain). *Geoderma*. **237-238**:270-282 (2015).
39. Chen, H., *et al.* Integration of an automated identification-quantification pipeline and statistical techniques for pyrolysis GC/MS tracking of the molecular fingerprints of natural organic matter. *Journal of Analytical and Applied Pyrolysis*. **134**:371-380 (2018).
40. Nowakowski, D.J., Woodbridge, C.R., and Jones, J.M. Phosphorus catalysis in the pyrolysis behaviour of biomass. *Journal of Analytical and Applied Pyrolysis*. **83**:197-204 (2008).
41. Klein, K., Gross-Schmölders, M., De la Rosa, J.M., Alewell, C., and Leifeld, J. Investigating the influence of instrumental parameters and chemical composition on pyrolysis efficiency of peat. *Communications in Soil Science and Plant Analysis*. **51**:1572-1581 (2020).
42. Pouwels, A.D., Eijkel, G.B., and Boon, J.J. Curie-point pyrolysis high-resolution gas chromatography–mass spectrometry of microcrystalline cellulose. *Journal of Analytical and Applied Pyrolysis*. **14**:237–280 (1989).
43. Hodgkins, S.B., *et al.* Tropical peatland carbon storage linked to global latitudinal trends in peat recalcitrance. *Nature Communications*. **9**:3640 (2018).
44. Caporaso, J.G., *et al.* Global patterns of 16S rRNA diversity at a depth of millions of sequences per sample. *Proceedings of the National Academy of Sciences*. **108**:4516-4522 (2011).
45. Thompson, L.R., *et al.* A communal catalogue reveals Earth's multiscale microbial diversity. *Nature*. **551**:457-463 (2017).
46. Callahan, B.J., *et al.* DADA2: High-resolution sample inference from Illumina amplicon data. *Nat Methods*. **13**:581-3 (2016).
47. Quast, C., *et al.* The SILVA ribosomal RNA gene database project: improved data processing and web-based tools. *Nucleic Acids Research*. **41**:D590-D596 (2012).
48. McMurdie, P.J. and Holmes, S. phyloseq: An R Package for Reproducible Interactive Analysis and Graphics of Microbiome Census Data. *PLOS ONE*. **8**:e61217 (2013).
49. James, G., Witten, D., Hastie, T., and Tibshirani, R. *An Introduction to Statistical Learning* (Springer New York, 2013).
50. Oksanen, J., *et al.* vegan: Community Ecology Package. *v 2.6-6.1. R package. Available: https://CRAN.R-project.org/package=vegan*. (2024).
51. Rodwell, J.S. *British plant communities: Volume 2, Mires and Heaths* (Cambridge University Press, 1998).
52. Wang, M., Moore, T.R., Talbot, J., and Riley, J.L. The stoichiometry of carbon and nutrients in peat formation. *Global Biogeochemical Cycles*. **29**:113-121 (2015).
53. Loisel, J., *et al.* A database and synthesis of northern peatland soil properties and Holocene carbon and nitrogen accumulation. *The Holocene*. **24**:1028-1042 (2014).
54. Crowe, S., Evans, M., and Allott, T. Geomorphological controls on the re-vegetation of erosion gullies in blanket peat: implications for bog restoration. *Mires & Peat*. **3** (2008).
55. Milner, A.M., *et al.* A regime shift from erosion to carbon accumulation in a temperate northern peatland. *Journal of Ecology*. **109**:125-138 (2021).
56. Biester, H., Knorr, K.H., Schellekens, J., Basler, A., and Hermanns, Y.M. Comparison of different methods to determine the degree of peat decomposition in peat bogs. *Biogeosciences*. **11**:2691-2707 (2014).
57. Darusman, T., Murdiyarso, D., Impron, and Anas, I. Effect of rewetting degraded peatlands on carbon fluxes: a meta-analysis. *Mitigation and Adaptation Strategies for Global Change*. **28**:10 (2023).
58. Evans, M.G., *et al.* Carbon Loss Pathways in Degraded Peatlands: New Insights From Radiocarbon Measurements of Peatland Waters. *Journal of Geophysical Research: Biogeosciences*. **127** (2022).
59. Strack, M., Zuback, Y., Mccarter, C., and Price, J. Changes in dissolved organic carbon quality in soils and discharge 10 years after peatland restoration. *Journal of Hydrology*. **527**:345-354 (2015).

60. Pinsonneault, A.J., Moore, T.R., Roulet, N.T., and Lapierre, J.-F. Biodegradability of Vegetation-Derived Dissolved Organic Carbon in a Cool Temperate Ombrotrophic Bog. *Ecosystems*. **19**:1023-1036 (2016).
61. Hamer, U. and Marschner, B. Priming effects of sugars, amino acids, organic acids and catechol on the mineralization of lignin and peat. *Journal of Plant Nutrition and Soil Science*. **165**:261-268 (2002).
62. Basiliko, N., Stewart, H., Roulet, N.T., and Moore, T.R. Do Root Exudates Enhance Peat Decomposition? *Geomicrobiology Journal*. **29**:374-378 (2012).
63. Jilling, A., Keiluweit, M., Gutknecht, J.L.M., and Grandy, A.S. Priming mechanisms providing plants and microbes access to mineral-associated organic matter. *Soil Biology and Biochemistry*. **158**:108265 (2021).
64. Ivanova, A.A., *et al.* Closely Located but Totally Distinct: Highly Contrasting Prokaryotic Diversity Patterns in Raised Bogs and Eutrophic Fens. *Microorganisms*. **8**:484 (2020).
65. Dedysh, S.N., *et al.* Acidobacteria in Fens: Phylogenetic Diversity and Genome Analysis of the Key Representatives. *Microbiology*. **91**:662-670 (2022).
66. Golovchenko, A.V., *et al.* Diversity and Functional Potential of Prokaryotic Communities in Depth Profile of Boreo-Nemoral Minerotrophic Pine Swamp (European Russia). *Forests*. **14**:2313 (2023).
67. Zubov, I., Shpanov, D., Ponomareva, T., and Aksenov, A. Bog bacterial community: data from north-western Russia. *Biodivers Data J*. **12**:e118448 (2024).
68. Carroll, R., Jeffries, T.C., and Reynolds, J.K. Sulfur Regulation on Microbial Biodiversity in a Montane Peatland. *European Journal of Soil Science*. **76**:e70045 (2025).
69. Oren, A. The Family Xanthobacteraceae in *The Prokaryotes: Alphaproteobacteria and Betaproteobacteria* (eds., E. Rosenberg, E.F. DeLong, S. Lory, E. Stackebrandt, and F. Thompson) 709-726 (Springer Berlin Heidelberg, 2014).
70. Leschine, S., Paster, B.J., and Canale-Parola, E. Free-Living Saccharolytic Spirochetes: The Genus Spirochaeta in *The Prokaryotes: Volume 7: Proteobacteria: Delta, Epsilon Subclass* (eds., M. Dworkin, S. Falkow, E. Rosenberg, K.-H. Schleifer, and E. Stackebrandt) 195-210 (Springer New York, 2006).
71. Aylward, F.O. and Santoro, A.E. Heterotrophic Thaumarchaea with Small Genomes Are Widespread in the Dark Ocean. *mSystems*. **5** (2020).
72. Biddle, J.F., *et al.* Heterotrophic Archaea dominate sedimentary subsurface ecosystems off Peru. *Proceedings of the National Academy of Sciences*. **103**:3846-3851 (2006).
73. Evans, P.N., *et al.* Methane metabolism in the archaeal phylum Bathyarchaeota revealed by genome-centric metagenomics. *Science*. **350**:434-438 (2015).
74. Yu, T., *et al.* Growth of sedimentary Bathyarchaeota on lignin as an energy source. *Proceedings of the National Academy of Sciences*. **115**:6022-6027 (2018).
75. Jayasekara, C., Leigh, C., Shimeta, J., Silvester, E., and Grover, S. Organic matter decomposition in mountain peatlands: effects of substrate quality and peatland degradation. *Plant and Soil*. **506**:639-654 (2025).
76. Preston, M.D., Smemo, K.A., McLaughlin, J.W., and Basiliko, N. Peatland microbial communities and decomposition processes in the James Bay Lowlands, Canada. *Frontiers in Microbiology*. **3**:70 (2012).
77. Russow, R., *et al.* Nitrate turnover in a peat soil under drained and rewetted conditions: results from a [15N] nitrate–bromide double-tracer study. *Isotopes in Environmental and Health Studies*. **49**:438-453 (2013).
78. Wiedermann, M.M., Kane, E.S., Potvin, L.R., and Lilleskov, E.A. Interactive plant functional group and water table effects on decomposition and extracellular enzyme activity in Sphagnum peatlands. *Soil Biology and Biochemistry*. **108**:1-8 (2017).
79. Straková, P., *et al.* Litter type affects the activity of aerobic decomposers in a boreal peatland more than site nutrient and water table regimes. *Biogeosciences*. **8**:2741-2755 (2011).
80. Elliott, D.R., Caporn, S.J.M., Nwaishi, F., Nilsson, R.H., and Sen, R. Bacterial and Fungal Communities in a Degraded Ombrotrophic Peatland Undergoing Natural and Managed Re-Vegetation. *PLOS ONE*. **10**:e0124726 (2015).

81. Andersen, R., Wells, C., Macrae, M., and Price, J. Nutrient mineralisation and microbial functional diversity in a restored bog approach natural conditions 10 years post restoration. *Soil Biology and Biochemistry*. **64**:37-47 (2013).
82. Mpamah, P.A., Taipale, S., Rissanen, A.J., Biasi, C., and Nykänen, H.K. The impact of long-term water level draw-down on microbial biomass: A comparative study from two peatland sites with different nutrient status. *European Journal of Soil Biology*. **80**:59-68 (2017).
83. Chen, X.*, et al.* Drainage-Driven Loss of Carbon Sequestration of a Temperate Peatland in Northeast China. *Ecosystems*. **27**:207-221 (2024).
