# Supplementary material for: Legacy of peatland erosion shapes microbial communities during recovery
Source: Commun Earth Environ. 2026 Jun 30;7(1):838. doi: 10.1038/s43247-026-03766-8 (PMC13683034; doi:10.1038/s43247-026-03766-8)
Supplement: Supplementary file 2 [file 43247_2026_3766_MOESM2_ESM.pdf]

Supplementary Tables

	Status (d.f.=2)			Depth (d.f.=1)			Status×Depth (d.f.=2)			Mixed model R^2	
**Variable**	***F*-stat.**	**d.d.f.**	***p***	***F*-stat.**	**d.d.f.**	***p***	***F*-stat.**	**d.d.f.**	***p***	**Conditional**	**Marginal**
Hemicellulose:Cellulose	6.91	37.0	0.003	2.77	37.0	0.104	0.66	37.0	0.525	-	0.342
Lignin:Polysaccharide	6.75	35.0	0.003	177.26	35.1	2.76×10^{-15}	58.61	35.1	6.59×10^{-12}	0.886	0.853
C:N	33.37	160.0	7.69×10^{-13}	22.33	160.0	5.00×10^{-6}	8.67	160.0	2.67×10^{-4}	-	0.339
pH	12.09	154.3	1.32×10^{-5}	3.68	154.6	0.057	1.48	153.8	0.231	0.376	0.268
Inorganic N	2.42	56.7	0.098	64.08	56.7	6.94×10^{-11}	19.66	56.7	3.27×10^{-7}	0.851	0.644
DOC	40.58	237.1	6.96×10^{-16}	4.16	237.4	0.042	3.23	237.1	0.041	0.749	0.174
CO_2	80.35	70.6	2.20×10^{-16}	234.64	72.0	2.20×10^{-16}	11.00	70.3	6.99×10^{-5}	0.942	0.932

Supplementary Table A | Statistical effects of condition, depth and their interaction on peat and porewater geochemistry, as tested by linear mixed modelling. Models also include a term to group samples by replicate as a random effect, while pH, inorganic N, DOC and CO_2 models include a term representing the sampling timepoint as a random effect. Where estimated variance of the random effect was negligible, the conditional model R^2 is not reported. Status was either Degraded; Restoration; or Natural-regeneration. d.d.f. = denominator degrees of freedom

Variable	Fixed effect	Estimate (β)	95% confidence intervals	
			Lower	Upper
Hemicellulose:Cellulose	ConditionN	0.55	0.25	0.84
	ConditionR	0.080	–0.22	0.38
	Depth	–0.00022	–0.0042	0.0037
	ConditionN:Depth	–0.0036	–0.0096	0.0024
	ConditionR:Depth	–0.0022	–0.0078	0.0035
Lignin:Polysaccharide	ConditionN	–0.12	–0.19	–0.046
	ConditionR	–0.12	–0.19	–0.051
	Depth	0.0011	0.00016	0.0020
	ConditionN:Depth	0.0077	0.0063	0.0092
	ConditionR:Depth	0.0011	–0.00021	0.0025
C:N	ConditionN	9.65	7.32	11.96
	ConditionR	5.31	3.18	7.44
	Depth	–0.0062	–0.025	0.013
	ConditionN:Depth	–0.089	–0.13	–0.046
	ConditionR:Depth	–0.0058	–0.033	0.021
pH	ConditionN	–0.31	–0.43	–0.18
	ConditionR	–0.25	–0.39	–0.12
	Depth	–0.000075	–0.0027	0.0026
	ConditionN:Depth	0.0029	–0.00043	0.0062
	ConditionR:Depth	0.0014	–0.0022	0.0049
Inorganic N (μM)	ConditionN	–8.57	−17.59	0.53
	ConditionR	–10.14	–19.53	–0.64
	Depth	0.82	0.61	1.03
	ConditionN:Depth	–0.85	–1.13	–0.57
	ConditionR:Depth	–0.14	–0.43	0.15
DOC (mM)	ConditionN	1.61	1.24	1.98
	ConditionR	0.52	0.13	0.91
	Depth	0.0079	−0.00031	0.016
	ConditionN:Depth	−0.011	−0.022	0.00026
	ConditionR:Depth	0.0010	−0.010	0.012
CO_2 (μM)	ConditionN	1720.28	1415.33	2028.34
	ConditionR	261.39	−71.96	596.68
	Depth	23.88	17.47	30.38
	ConditionN:Depth	13.53	5.41	21.56
	ConditionR:Depth	−3.96	−12.56	4.60

Supplementary Table B | Effect sizes and 95% confidence intervals of fixed-effects from linear mixed modelling. Degraded was used as the reference peatland condition in model fitting; therefore, ConditionN and ConditionR represent contrasts between Natural regeneration or Restoration relative to Degraded peatland, respectively.

Supplementary Figures

Supplementary Figure A | Cover of bare peat and vegetation across the selected study locations at Waun Fignen Felen. Photographs of the five spatial replicates within each state are shown in **a** (see Fig. 1 for locations map; PWE = porewater equilibrator samplers); plant ID shown in **b**. Plant identification survey took place on 5 July 2022. Plant percentage cover was estimated within a 2×2 m quadrat centred around the sampling locations (with bare peat included as a cover type), from which relative abundance values were calculated. Identification relied on field observation and photographs, with *Sphagnum* ID confirmed by examination with a microscope. Ellenberg moisture values from Hill et al.[1-2].

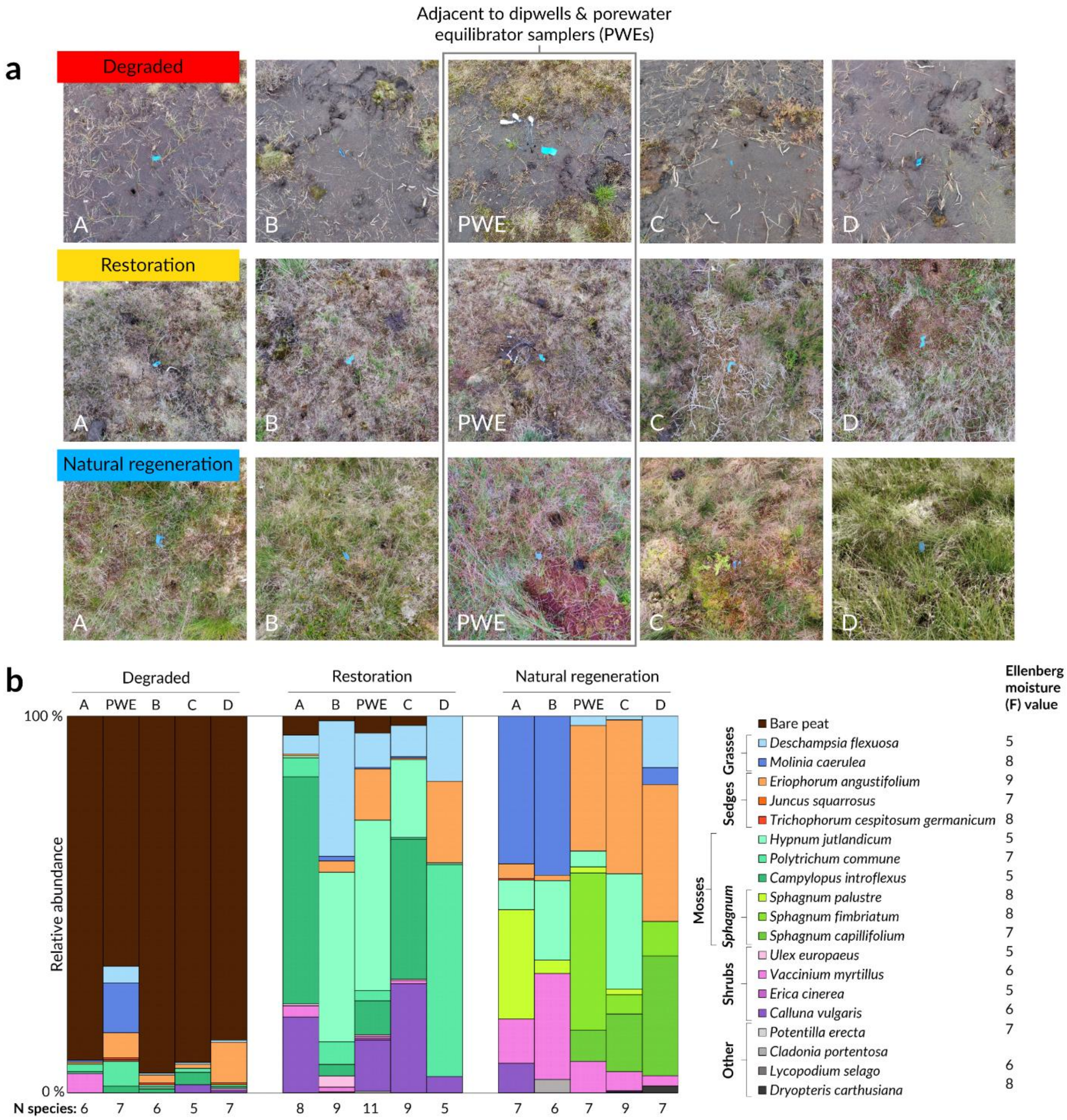

Supplementary Figure B | Water table response to precipitation and drought. Logged water table positions in select periods of high rainfall and drought across the three study conditions at Waun Fignen Felen. Bars at base represent daily rainfall totals recorded at the site (right-hand y-axis).

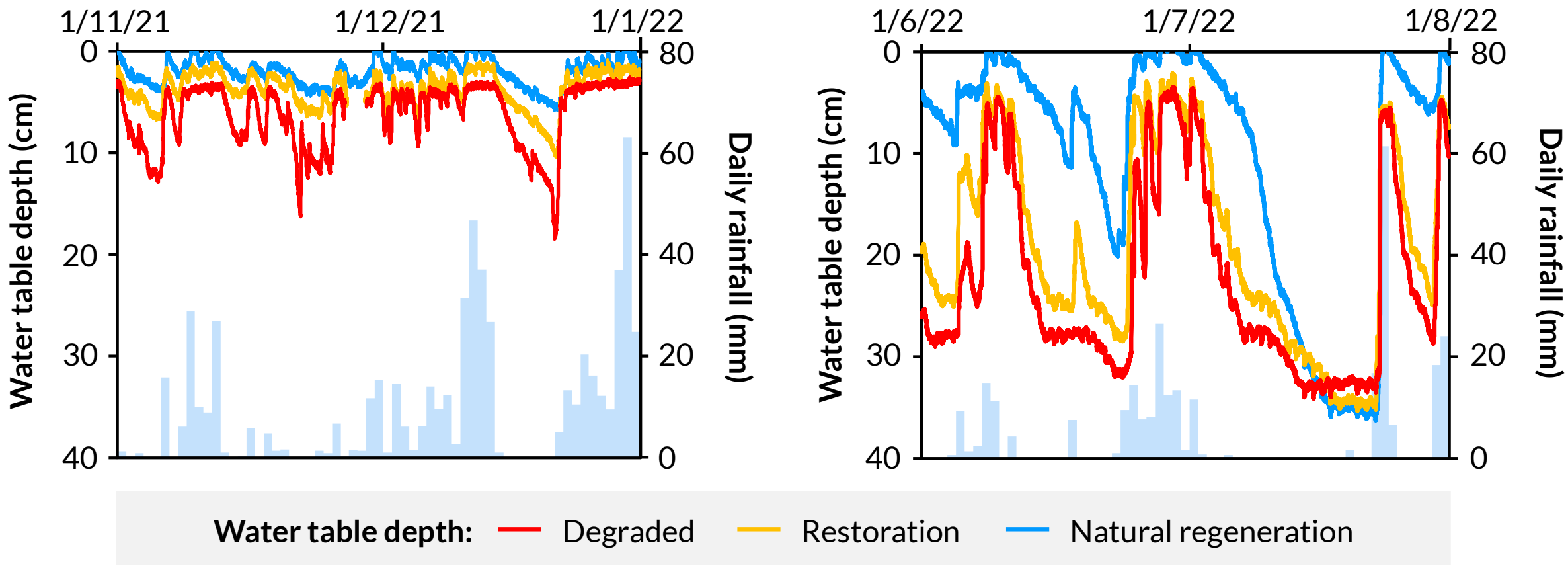

Supplementary Figure C | Hydraulic conductivity at Waun Fignen Felen. K_{sat} values estimated using the slug-withdrawal method, presented on a logarithmic scale with repeat measurements shown. While nominal depths are plotted, note the K_{sat} values represent overall conductivity for the ±10 cm region in contact with the piezometer screen. Data collected on 27 January 2022, while peat was under saturated conditions in all cases.

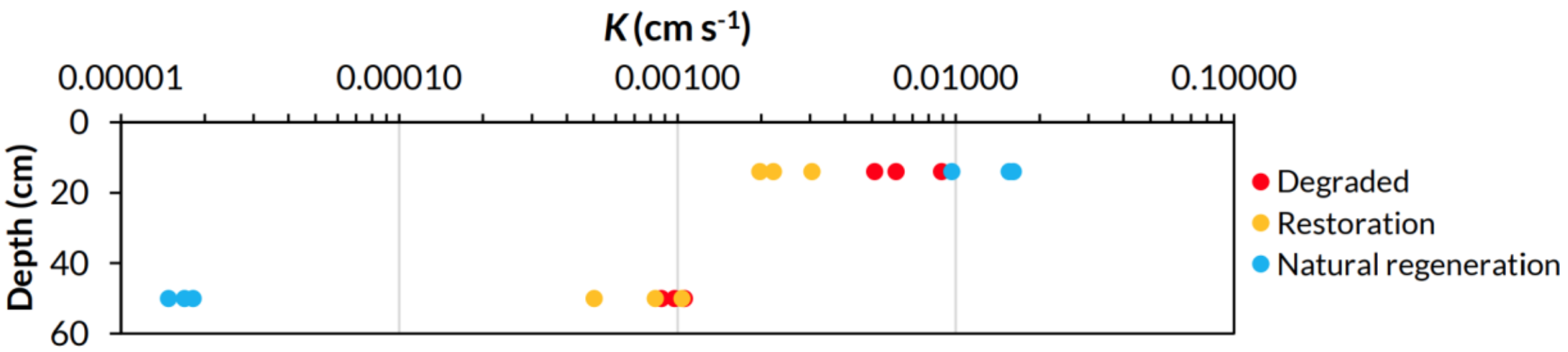

Supplementary Figure D | Depth profiles of dissolved methane in porewater at Waun Fignen Felen. Points represent mean values with standard deviation shown by bars, data was collected on the 4 sampling occasions between May-October 2022.

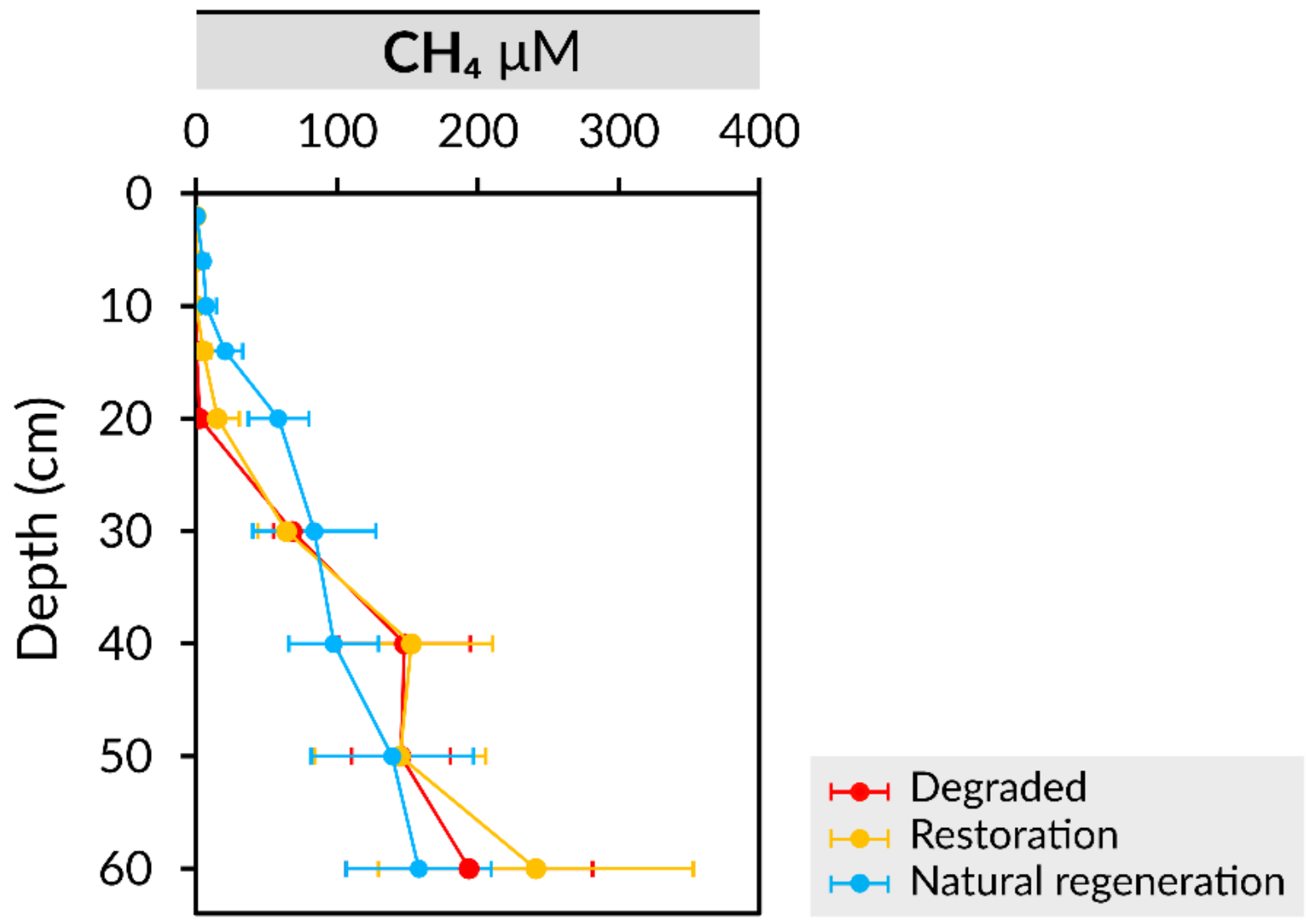

Supplementary Figure E | Depth profiles of dissolved ammonium, and total oxides of nitrogen (nitrate + nitrite). Points represent mean values with standard deviation shown by bars, data was collected on 6 sampling occasions between September 2021-October 2022 (13/09/2021, 09/12/2021, 03/03/2022, 12/04/2022, 25/08/2022 & 12/10/2022).

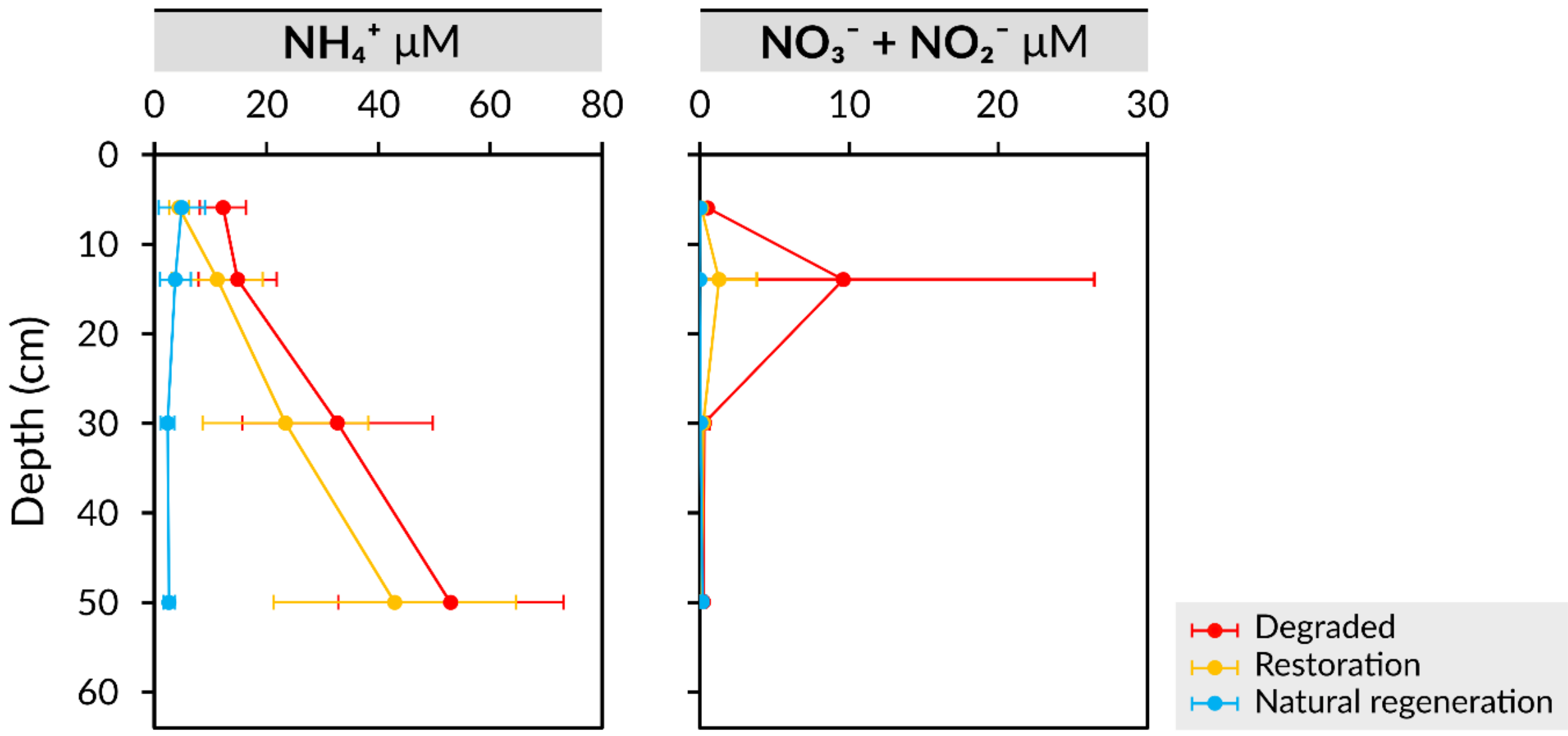

Supplementary Notes

1. Note on Figure 1 in main text. Figure 1a in the main text was produced using QGIS software and spatial data on estimated peatland extent from the PEATMAP project [3]. Other panels were produced entirely by the authors; photographs of the site in panels c-e were taken by FRH.

Supplementary References

1. Hill, M.O., Mountford, J.O., Roy, D.B., and Bunce, R.G.H., Ellenberg's indicator values for British plants. ECOFACT Volume 2 Technical Annex. 1999, Institute of Terrestrial Ecology, Cambridgeshire, UK.
2. Hill, M.O., Preston, C.D., Bosanquet, S.D.S., and Roy, D.B., BRYOATT. Attributes of British and Irish Mosses, Liverworts and Hornworts. 2007, Centre for Ecology and Hydrology, Cambridgeshire, UK.
3. Xu, J., Morris, P.J., Liu, J., and Holden, J. PEATMAP: Refining estimates of global peatland distribution based on a meta-analysis. University of Leeds. [Dataset]. (2017).
